# Tiny messengers, big impact: unlocking the power of extracellular vesicles in neonatal health and disease. a systematic review

**DOI:** 10.3389/fimmu.2026.1848637

**Published:** 2026-06-22

**Authors:** Alexandra Lianou, Andreas G. Tsantes, Sotirios P. Fortis, Effie G. Papageorgiou, Anastasios G. Kriebardis, Panagiotis G. Zoumpoulakis, Zoi Iliodromiti, Theodora Boutsikou, Evangelos Terpos, Maria Gavriatopoulou, Nicoletta Iacovidou, Rozeta Sokou

**Affiliations:** 1 Neonatal Intensive Care Unit, “Aghios Panteleimon” General Hospital of Nikea, Piraeus, Greece; 2 Microbiology Department, “Saint Savvas” Oncology Hospital, Athens, Greece; 3 Laboratory of Haematology and Blood Bank Unit, “Attikon” Hospital, School of Medicine, National and Kapodistrian University of, Athens, Athens, Greece; 4 Laboratory of Reliability and Quality Control in Laboratory Hematology (HemQcR), Department of Bio-Medical Sciences, School of Health & Caring Sciences, University of West Attica (UniWA), Athens, Greece; 5 Laboratory of Chemistry, Analysis and Design of Food Processes, Department of Food Science and Technology, University of West Attica, Athens, Greece; 6 National Hellenic Research Foundation, Institute of Biology, Medicinal Chemistry and Biotechnology, 48 Vassileos Constantinou, Athens, Greece; 7 Neonatal Department, National and Kapodistrian University of Athens, Aretaieio Hospital, Athens, Greece; 8 Department of Clinical Therapeutics, School of Medicine, National and Kapodistrian University of Athens, Athens, Greece

**Keywords:** exosomes, extracellular vesicles, hemostasis, microparticles, neonates

## Abstract

**Objective:**

Extracellular vesicles (EVs) are cell-derived, membrane-bound particles that have gained increasing attention for their roles in pathophysiology, diagnostics, and targeted therapies. This review aimed at systematically collecting and critically synthesizing current evidence on EVs derived from neonatal biological fluids, focusing on their involvement in neonatal health and disease and their potential diagnostic and prognostic value.

**Methods:**

A systematic literature review was conducted in PubMed and Scopus databases between August and November 2025. The study protocol was registered in PROSPERO (CRD420251151417).

**Results:**

Following a PRISMA-compliant search strategy, 43 predominantly observational studies were included, encompassing term and preterm neonates with small-to-moderate sample sizes and heterogeneous clinical settings. EVs were isolated from multiple biological matrices, most frequently umbilical cord and peripheral blood, with sampling mainly performed at birth or during early postnatal life. Overall, the findings demonstrate the consistent presence and biological activity of EVs across diverse neonatal conditions, supporting their potential role as pathophysiological mediators and candidate biomarkers.

**Conclusions:**

Although EVs appear to actively participate in key neonatal processes, the available evidence remains limited and methodologically heterogenous. Larger, standardized studies are required to clarify their biological functions and clinical utility.

**Systematic Review Registration:**

https://www.crd.york.ac.uk/PROSPERO/view/CRD420251151417, identifier CRD420251151417.

## 1 Introduction

Extracellular vesicles (EVs) constitute a distinct group of cellular components of great scientific interest, as they are recognized as primary agents of cellular life and activity (1). EVs are membrane bound nanostructures, enclosed in a lipid bilayer, which are synthesized and secreted by various cell types in response to cellular stimulation (activation, inflammation, antibody binding, etc.), to invading pathogens, or during programmed cell death (apoptosis) (1, 2). EVs belong to the broader category of extracellular particles (EPs). EPs include all cell-derived entities found in the extracellular milieu and comprise both vesicular and non-vesicular particles. Non-vesicular extracellular particles (NVEPs) are composed of cell-derived components of one or more molecular classes (e.g., proteins, nucleic acids) and lack a lipid bilayer membrane (3).

Despite their signaling functions being historically underrecognized, the procoagulant properties of EVs—initially described as “platelet dust”—were identified early on. Current evidence highlights their critical role as bioactive mediators involved in the transfer of diverse cellular components, with their biological functions closely reflecting their cellular origin (4). EVs cargo, includes proteins (e.g. cell surface receptors, signaling proteins, transcription factors, enzymes, and extracellular matrix proteins), procoagulant phospholipids such as phosphatidylserine (PS), and nucleic acids (including miRNA (microRNA), mRNA, and DNA), which can be transferred among cells, thereby mediating intercellular communication. Thus, they facilitate complex cellular interactions and mediate a wide range of biological processes (5, 6).

EVs display considerable heterogeneity in size, arise from diverse cellular and microbial sources, and exhibit diverse morphologies that reflect the stimulus and mechanism underlying their biogenesis and release (7). Small EVs (SEVs) -including exosomes- typically <200 nm in diameter, originate mainly from the inward budding of multivesicular bodies and contribute to intercellular signaling. In contrast, large EVs (LEVs), which can reach approximately 1 μm in size and include microvesicles or microparticles (MPs), arise from outward budding of the plasma membrane and are frequently implicated in inflammatory and coagulation pathways. Platelet-derived EVs (PDEVs) constitute a substantial proportion of the circulating pool (7). The procoagulant properties of EVs largely stem from the externalization of PS, which offers a catalytic surface for coagulation enzyme complexes, as well as from tissue factor (TF) present on EVs released by endothelial cells and monocytes (1, 8, 9). The International Society for Extracellular Vesicles (ISEV) updated the minimal information for studies of EVs (MISEVs) guidelines to enhance the quality, consistency, and reproducibility of EV research. These recommendations emphasize transparent reporting of sample collection, isolation, and storage procedures, as well as thorough EV characterization using established EV markers and a minimum of two complementary single-particle analytical techniques (3).

EVs participate in numerous physiological and pathological processes and are increasingly recognized for their diagnostic and therapeutic potential, especially in adults. Tumor-derived EVs actively remodel the tumor microenvironment and carry stable protein and RNA cargo detectable in blood and other biofluids, enabling liquid biopsy approaches for early diagnosis, risk stratification, and prediction of treatment response (10). Disease-specific EV molecular signatures in biofluids also reflect pathological states in cardiovascular disease, diabetes, neurodegeneration, and sepsis, supporting longitudinal disease monitoring (5). Therapeutically, EVs can be targeted through elimination of circulating EVs, inhibition of EV release, or blockade of cellular uptake, while engineered EVs function as biocompatible nanocarriers for targeted drug and gene delivery with low immunogenicity (5).

Importantly the specific role of EVs within the unique physiological landscape of the neonate remains under-explored. It is known that EVs are present across various neonatal biological fluids, such as umbilical cord blood (UCB), peripheral blood, and meconium and participate in essential processes like hemostatic adaptation and fetal-maternal signaling (11). Additionally, experimental studies increasingly support EVs as promising therapeutic tools for complications of prematurity. Mesenchymal stem cell–derived EVs showed beneficial effects in preclinical models of bronchopulmonary dysplasia (BPD) and necrotizing enterocolitis (NEC), while microglia-derived EVs enriched in miR-24-3p attenuate retinal injury in models of retinopathy of prematurity (ROP) (12).

Although the role of EVs was described in various neonatal diseases, it should be emphasized that the number of available studies providing data on EVs specifically in neonates remains very limited compared to adults. Moreover, neonates constitute a unique and biologically distinct population, therefore data derived from adult studies cannot be directly extrapolated to neonates. EVs hold considerable potential for elucidating mechanisms of neonatal health and disease, as well as advancing diagnostic and therapeutic applications. However significant gaps persist regarding the developmental trajectory of EV formation across gestation and how their molecular cargo dynamically influences recipient cell functions in a tissue-specific manner. Despite growing interest, studies on EVs in neonatal diseases remain scarce, heterogeneous, and largely exploratory. As a result, the clinical applicability, diagnostic utility, and therapeutic potential of EVs in neonatal care remain insufficiently defined. Therefore, the present systematic review aims to gather and qualitatively present the existing evidence on the biological role and translational potential of EVs within the distinct neonatal population.

## 2 Materials and methods

### 2.1 Protocol registration

A systematic review was conducted following a predefined protocol aligned with PRISMA (Preferred Reporting Items for Systematic Reviews and Meta-Analyses) guidelines. The research protocol was registered on PROSPERO on the 19^th^ of September 2025 (CRD420251151417). The research strategy adapted to identify, evaluate and interpret the available studies relevant to our investigational objective followed the guidelines of the PRISMA Statement (13). (**Supplementary File 1**).

### 2.2 Eligibility criteria

Investigational query: Studies containing original data regarding the role and clinical application of EVs as novel agents in the understanding of neonatal physiology/pathophysiology, as putative diagnostic/prognostic biomarkers of neonatal health and disease and as putative therapeutic mediators for the neonatal population.

The study population included neonates term and preterm during the first moth of life. The intervention included the identification of EVs and their subtypes (e.g. microparticles, exosomes, microvesicles) in terms of size, concentration, and their cargo (e.g. miRNAs, proteins etc.) from samples of neonatal biofluids. The following study types, with global regional distribution were included: Randomized clinical trials (RCTs), cohort studies (prospective or retrospective), observational studies irrespective of the number of patients included or the number of study centers, case-control studies, cross-sectional studies and letters to the editor containing original data. There were no geographical or time restrictions regarding the search strategy and study selection.

### 2.3 Exclusion criteria

Studies including subjects other than neonates in the first moth of life, without separately exhibiting data concerning neonates, animal and experimental studies and studies not in English language were not included in our review. Articles classified as reviews (systematic or narrative), meta-analyses, and conference proceedings were not eligible for inclusion in this study.

### 2.4 Study outcomes

Primary outcomes included i) the characterization of EVs during extrauterine adaptation, ii) the role of EVs as key elements in the physiology and pathophysiology of various neonatal conditions. iii) the role of EVs as diagnostic, prognostic or therapeutic tools of various neonatal disorders.

Secondary outcomes included i) The identification of EV-cargo in healthy neonates ii) The characterization and the role of EV-cargo in various neonatal disorders.

### 2.5 Search strategy

A search algorithm was conducted and applied to PubMed and Scopus databases until August 7, 2025, and the search was then updated on November 10, 2025. The keyword combination used was: “extracellular vesicles”, “extracellular vesicle”, “microvesicles”, “microvesicle”, “microparticles”, “microparticle”, “neonate”, “neonates”, “neonat*, “newborn”, “newborns”, “perinatal” utilizing Boolean logical operators (AND, OR) for the search algorithm conduction. All mined references were entered in Rayyan online platform for screening. (**Supplementary Text File 1**).

### 2.6 Data extraction and synthesis

Data were extracted from full texts of the included studies and incorporated into Excel. The procedure was carried out independently by two reviewers, with any disagreements or queries resolved through consensus. In addition to the primary and secondary outcomes, information on study characteristics, and other outcomes assessed in the articles, were also extracted. Owing to substantial heterogeneity in population characteristics, methods of EVs evaluation, and clinical conditions being studied, meta-analysis was considered infeasible.

## 3 Results

### 3.1 Study selection

The first database search conducted on 7 August 2025 yielded 1,426 records, of which 480 we removed by a single reviewer (A.L) as duplicates. The remaining 952 records underwent independent title and abstract screening by two different reviewers (A.L. and R.S.) and resulted in the exclusion of 608 articles that did not meet the scope of the review or satisfied predefined exclusion criteria. Consequently, 344 articles proceeded to full-text assessment. Review articles (n = 119) were excluded at this stage, and detailed evaluation of the remaining 225 studies led to the inclusion of 33 eligible articles.

An updated database search using the same strategy, was performed between August 7 and November 10, 2025, and provided 67 additional records. Following duplicate removal of 16 records, 51 articles were screened by title and abstract with 26 excluded. The remaining 25 articles underwent full-text evaluation and only 1 met the inclusion criteria.

To minimize the risk of missing relevant studies a systematic snowballing approach was applied. Reference lists of all included articles and relevant systematic reviews, were screened. Titles referring to EVs, microparticles, exosomes or microvesicles in neonates were selected for full-text review. Potentially eligible articles were assessed by two independent reviewers (A.L and R.S.) for final inclusion resulting to 9 studies that met the inclusion criteria.

**Figure 1:**
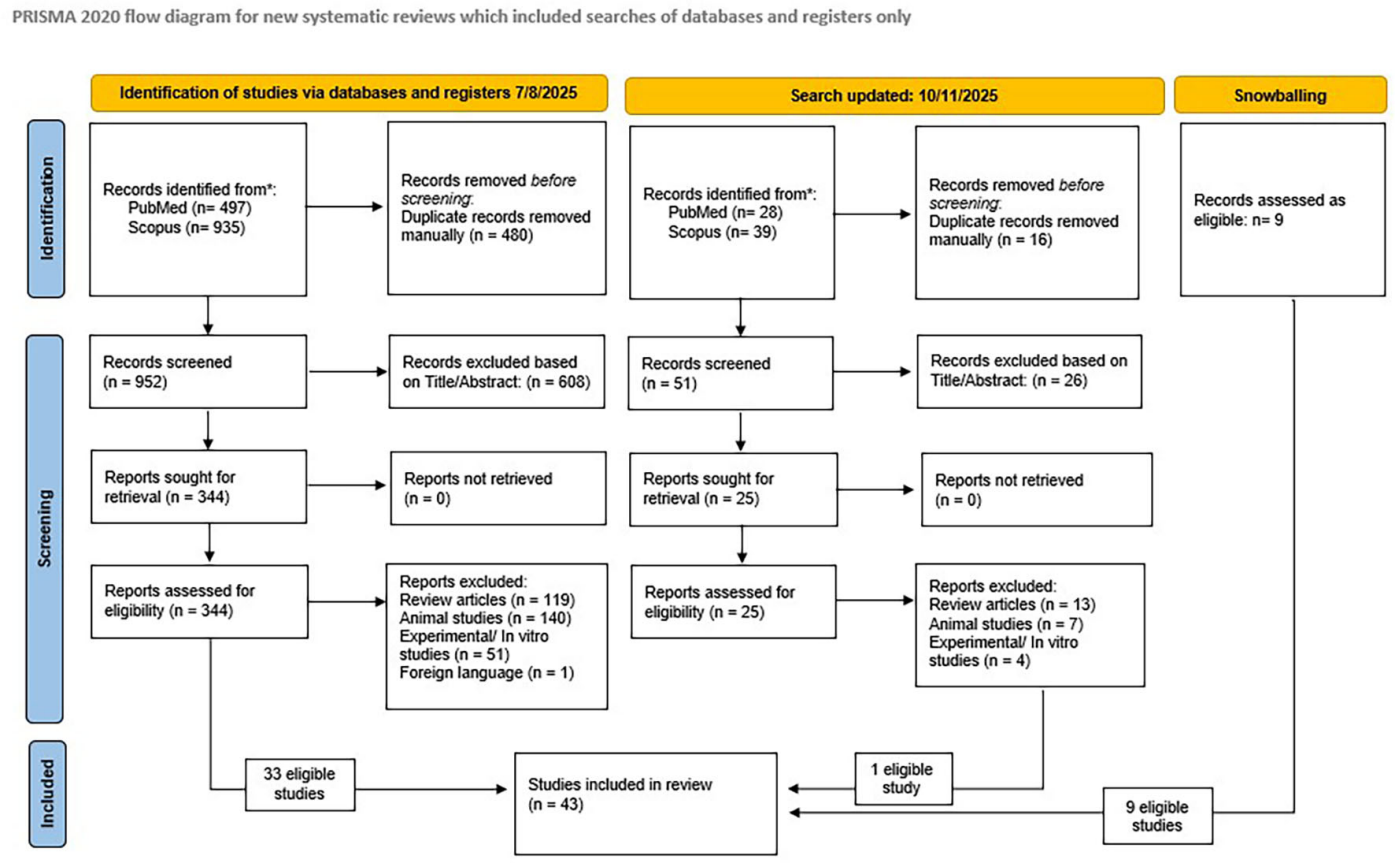
PRISMA 2020 Flow chart for systematic reviews. Schematic representation of study selection process.

Overall, a total of 43 studies were included in the present systematic review. Disagreements throughout the screening and selection process were resolved through discussion or, when necessary, consultation with a third reviewer (A.G.T.). **Figure 1** presents the PRISMA 2020 flow diagram summarizing the study selection process.

### 3.2 Overview of the included studies

#### 3.2.1 Study characteristics

**Figure 2:**
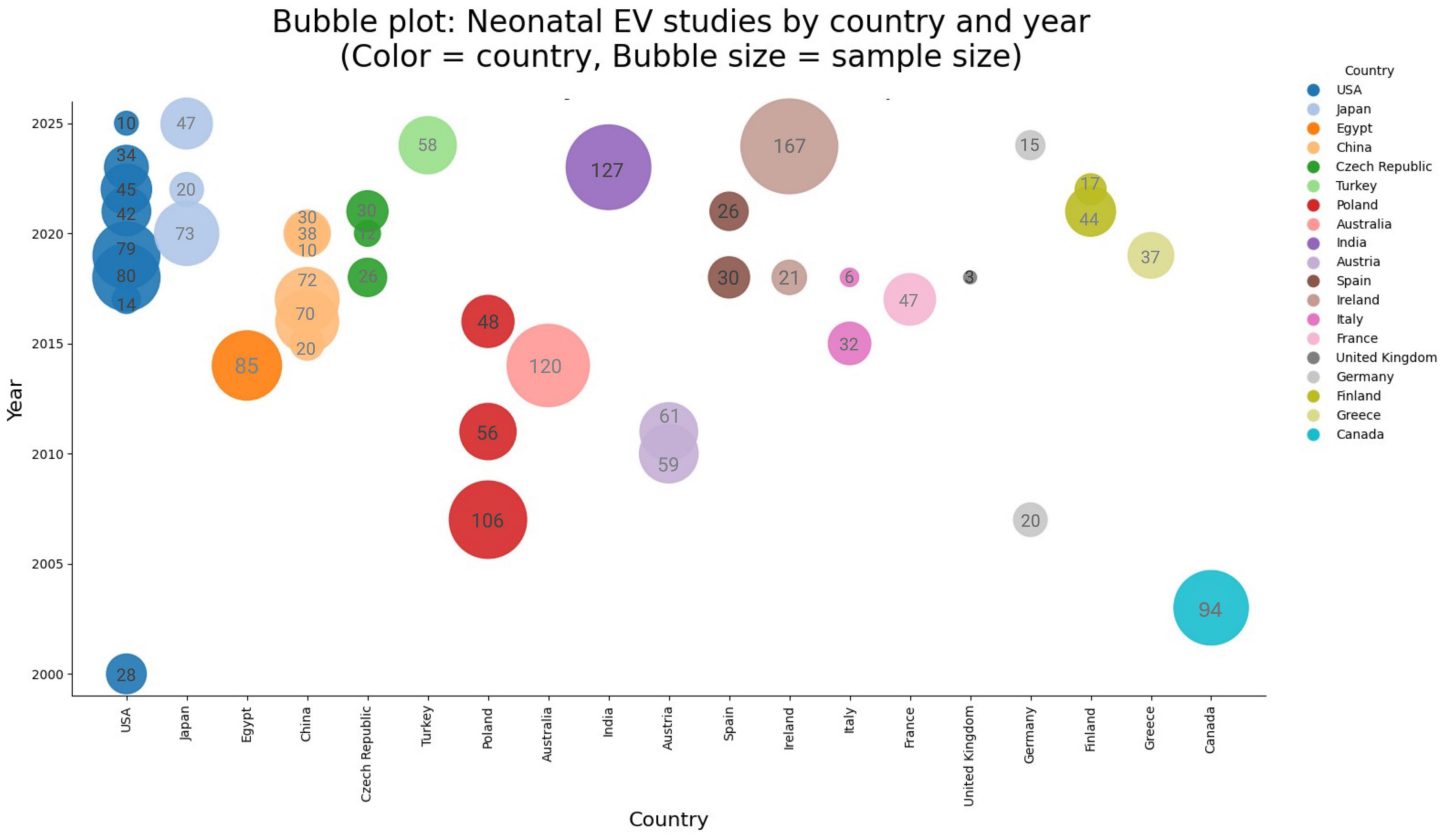
Schematic representation of the number of studies assessing EVs by country and year. The figure highlights the rising scientific interest regarding EVs in neonates in the past few years. Numbers in the bubbles represent sample sizes. (Created with ChatGPT 5.3 based on the data from **Table 1**).

The studies included in this review, exhibited a worldwide distribution and highlighted the increasing scientific interest that EVs have gained in the field of neonatology over the past few years (**Figure 2**). The geographical distribution included studies from: the United states of America (8 records) (14–21), Canada (1 record) (22), Europe (22 records) (8, 9, 11, 23–41), Egypt (1 record) (42), India (1 record) (43), China (6 records) (44–49), Japan (3 records) (50–52), Australia (1 record) (53).

The selection process and data extraction revealed substantial heterogeneity across the included studies with respect to study objectives, study populations, assessed outcomes, types of EVs, timing of assessment, as well as the biological matrices analyzed and analytical methodologies applied. Due to this heterogeneity, a meta-analysis was considered methodologically inappropriate. Therefore, a qualitative synthesis of the available evidence was performed.

A total of 43 studies were included in the qualitative synthesis of this review. All included studies were observational studies some of which containing additional experimental/laboratory investigations. The majority of the studies were cross sectional studies (26 studies), 8 were prospective cohort studies, 4 were case-control studies, 4 were experimental/laboratory observational studies and 1 was case series with presentation of original data. Of the 43 included studies, 3 studies (26, 35, 53) were letters to the editor containing original data that serve the scope of this review. The research provided no RCTs on EVs as therapeutic agents in the neonatal population and studies regarding the therapeutic potential of EVs were all in experimental stages and did not meet the inclusion criteria of this systematic review.

All studies included neonates with samples eligible for the assessment of EVs and their cargo. The studied populations included neonates with gestational ages spanning from extreme prematurity to term delivery. Several studies incorporated maternal, adult, children or term-born control groups for comparison. In 11 studies, neonates, children and adults were included, but information was provided separately for each group. Three studies assessed EVs in neonates prospectively including timepoints that exceed the neonatal period of the first 28 days of life (15, 40, 50). However, data are presented separately for each time point. Sample sizes were generally small to moderate, ranging from approximately 10 to 170 participants, with substantial heterogeneity in gestational ages and clinical contexts (**Table 1**).

**Table 1:** Overview of the included studies.

Publication	Year	Country	Study design	Study population	Clinical field	Timepoint of sampling	Biofluid	Origin of EVs	Aim of the study
Alhamdan F. et al. (21)	2025	USA	Prospective cohort study	Total = 10 neonates, N = 5 neonates with OD, N = 5 neonates with NOD	CHD / Postoperative outcomes	T0 – Before surgery begins T1 – During the rewarming phase of CPB T2 –Upon admission to the ICU after surgery T3 – On postoperative day 1	Plasma (Peripheral blood)	Not recorded	To investigate whether plasma EV-derived miRNAs differ between neonates and infants with and without postoperative organ dysfunction after congenital heart surgery, and to elucidate the related biological mechanisms.
Vítková V. et al. (8)	2018	Czech Republic	Case-control study	Total= 26, N = 13 neonates on ECMO, N = 14 healthy neonates	Critically ill neonates	Controls: 48–72 h of life ECMO group: 36–350 h after ECMO initiation	Plasma (Peripheral blood)	Endothelial cells	To investigate whether endothelial microvesicles and soluble endothelial markers in plasma reflect inflammation and endothelial injury in critically ill newborns on ECMO, compared with healthy term newborns.
Ohta M. et al. (50)	2022	Japan	Prospective cohort study	Total= 20 preterm infants	Extrauterine adaptation	At birth and at 2, 4, 8, 24, and 48 weeks of age.	Serum (Peripheral blood)	Not determined	To assess the chronological and gestational age–related changes in serum exosome concentrations in preterm infants during the first year after birth.
Murphy C.A. et al. (11)	2024	Ireland	Prospective cohort study	Total=167, N = 101 preterm, N = 66 term	Extrauterine adaptation	At birth (UCB) Day 1 (0–24 hours of life) Day 3 (Preterm: 48–72 h / Term: 25–96 h) Day 14–21 (preterm only)	Plasma (UCB & Peripheral blood)	Platelets, Endothelial cells Leukocyte cells	Characterize how circulating extracellular vesicles change during early postnatal adaptation in preterm infants, and compare these patterns with those in term infants.
Awad H.A. et al. (42)	2014	Egypt	Prospective cohort study	Total= 85 neonates, N = 45 ABO-HDN, N = 20 Rh-HDN, N = 20 Control	HDN	Pre and post exchange transfusion and/or phototherapy	Plasma (Peripheral blood)	Endothelial cells	To investigate whether circulating CD144^+^ endothelial microparticles reflect endothelial injury in neonatal ABO haemolytic disease and correlate with disease severity.
Zhu X.J. et al.	2017	China	Prospective cohort study	Total= 72, N = 29 ABO-HDN, N = 22 Rh-HDN, N = 21 Controls	HDN	Pre and post exchange transfusion and/or phototherapy	Plasma (Peripheral blood)	Endothelial cells	To determine whether circulating CD144^+^ endothelial microparticles can serve as a prognostic biomarker in neonates with hemolytic disease of the newborn (especially ABO HDN) in a Chinese population.
Hujacova A. et al. (26)	2020	Czech Republic	Pilot comparative study	Total= 12, N = 5 preterm infants, 7 term infants	Hemostasis	At birth	Plasma (UCB)	Platelets	To assess the effect of pre-analytical factors (delay in processing and freezing) on platelet EV detection in cord blood, and to compare platelet EV characteristics between term and preterm newborns.
Korbal P. et al. (35)	2016	Poland	Pilot comparative study	Total= 48, N = 23 preterm infants, 25 term infants	Hemostasis	At birth	Plasma (UCB)	Not determined	To investigate the levels of TF and TF-bearing microparticles in the UCB of preterm and term neonates, and to examine their relationship with GA and BW.
Schweintzger S. et al. (23)	2011	Austria	Cross-sectional study	Total= 59, N = 31 Term neonates, N = 28 adults	Hemostasis	Neonates: at birth, Adults: single timepoint	Plasma (UCB)	Not determined	To investigate whether microparticles in newborn cord plasma differ from those in adult plasma in terms of concentration, procoagulant activity, and effect on thrombin generation.
Uszynski M. et al. (9)	2011	Poland	Cross-sectional study	Total= 56, N = 28 mother-infant pairs	Hemostasis	At bith	Plasma (UCB)	Not determined	To investigate the presence and concentration of microparticles in cord blood, compare them with maternal blood, and assess their relationship with TF and TFPI in fetal hemostasis
Wasiluk A. et al. (36)	2007	Poland	Cross-sectional study	Total= 106, N = 51 preterm neonates, N = 55 term neonates	Hemostasis	At birth	Plasma (UCB)	Platelets	To determine whether the percentage of PDMP reflects platelet activation in preterm newborns, and whether the platelet count is related to the percentage of PDMP.
Michelson A.D. et al. (20)	2000	USA	Cross-sectional study	Total=28, N = 7 Term neonates, N = 8 Preterm neonates, N = 13 adults	Hemostasis	Neonates: at birth, Adults: single timepoint	Plasma (UCB & Peripheral blood)	Platelets	To develop a flow cytometric method to assess PDMP procoagulant activity in whole blood and compare PDMP/platelet Factor V/Va binding between preterm, term neonates, and adults, exploring the role of factor V deficiency in preterm neonates
Peñas-Martínez J. et al. (38)	2021	Spain	Cross-sectional study	Total= 26, N = 14 Term neonates, N = 12 adults	Hemostasis	Neonates: at birth, Adults: single timepoint	Plasma (UCB & Peripheral blood)	Not determined	To investigate differences between neonatal and adult plasma-derived exosomes, focusing on their size, protein content, and proteomic composition, in order to understand age-related variations in exosome biology and their potential implications for neonatal hemostasis and transfusion outcomes.
Schmugge M. et al. (22)	2003	Canada	Partly Cross-sectional study, Partly longitutional	Total= 94, N = 38 Term neonates with UCB samples, N = 19 neonates with peripheral blood samples 2–3 DOL (15 of which were paired repeat samples from the UCB group, 4 were sampled only once), N = 15 Children, N = 22 Adults	Hemostasis	Neonates: At birth, 2–3 DOL Children & Adults: Signle timepoints	Plasma (UCB & Peripheral blood)	Platelets	To investigate VWF binding to platelets and its relationship with platelet activation in healthy neonates compared to children and adults.
Schweintzger S. et al. (24)	2010	Austria	Cross-sectional study	Total= 61, N = 31Term neonates, N = 28 adults	Hemostasis	At bitrh	Plasma (UCB & Peripheral blood)	Platelets	To investigate if newborn cord plasma versus adult plasma contains higher levels of microparticles able to support coagulation.
Karlaftis V. et al. (53)	2014	Australia	Prospective cohort study	Total =120, N = 10 term neonates, N = 80 children, N = 20 adults	Hemostasis	Neonates: 1 & 3 DOL, Children & Adults: Single timepoint	Plasma (Peripheral blood)	Not determined	To asses the age-specific differences in circulating MPs in healthy neonates, children and adults and to investigate whether microparticle-related procoagulant phospholipid activity changes with age.
Yücesoy E. et al. (39)	2024	Turkey	Prospective cohort study	Total = 58, N = 33 Non hemolytic hyperbilirubinemia cases, N = 25 Controls	Hyperbilirubinemia	Pre and post phototherapy (Cases), Single timepoint (Controls)	Plasma (Peripheral blood)	Endothelial cells, Platelets	To evaluate the effect of nonhemolytic pathologic hyperbilirubinemia and phototherapy on total apoptotic, platelet-derived, endothelial-derived, and TF-positive apoptotic MP levels in term neonates.
Wang D. J. et al. (46)	2016	China	Experimental/Laboratory Observational study	Total= 70 UCB samples from term pregnancies	Lactation	At birth	UCB (No specific information)	Not derermined	To investigate the expression of lactation-related microRNAs in microvesicles from human UCB immediately after delivery, and to explore whether these microvesicles can influence milk protein (β-casein) production in mammary epithelial cells.
Turunen J. et al. (27)	2021	Finland	Cross-sectional study	Total= 44 Term neonates, N = 23 neonates (vaginal deliveries), N = 21 neonates (C-sections)	Microbiome	Meconium: within first 24h of life, Placenta: Immediately after delivery. Amniotic fluid: at delivery	First pass Meconium, Amniotic fluid, Placenta	Not determined (possibly bacterial)	To critically evaluate the fetal microbiome concept by analyzing microbiomes of placenta, amniotic fluid, and first-pass meconium, and explore whether meconium microbiota may arise from perinatal colonization or bacterial EV transfer.
Turunen J. et al. (27)	2022	Finland	Cross-sectional study	Total= 17 Term neonates, N = 5 neonates (C-section), N = 6 neonates (vaginal delivery without intrapartum antibiotics), N = 6 neonates (vaginal delivery with intrapartum antibiotic exposure)	Microbiome	Within first 24h of life	First pass Meconium	Bacterial	To identify and characterize bacterial extracellular vesicles in first-pass meconium and explore whether their composition differs by delivery mode and intrapartum antibiotic exposure.
Galley J.D. et al. (14)	2021	USA	Prospective cohort study	Total= 42, N = 9 medical NEC cases, N = 13 surgical NEC cases, N = 8 non-NEC sepsis cases, N = 12 Controls	NEC	T1: Within 24 hours of enrollment (immediately after diagnosis) T2: 2 to 5 days after enrollment (during disease) T3: 6 to 14 days after enrollment (recovery phase)	Urine	Not determined	To determine whether urine-derived EV miRNAs are differentially expressed in infants with NEC compared to infants with sepsis and healthy controls, and whether they could serve as diagnostic biomarkers for differentiating NEC from other conditions.
Keller S. et al. (41)	2007	Germany	Experimental/Laboratory Observational study	Total= 20, N = 5 neonates, N = 11 adults, N = 4 women undergoing amniocentisis	Neonatal physiology	Shortly after birth (not specified)	Urine	Kidneys	To establish CD24 as a marker of exosomes and characterize exosomes in urine and amniotic fluid, including demonstrating fetal origin of amniotic exosomes
Huang S. et al. (44)	2020	China	Cross-sectional study	Total= 10, N = 5 Term neonates, N = 5 adults	Neonatal physiology	Neonates: At birth, Adults: single timepoint	Plasma (UCB & Peripheral blood)	Not determined	To assess differential expression profiles of miRNAs in exosomes derived from human UCB & peripheral blood.
Xagorari A. et al. (31)	2019	Greece	Cross-sectional study	Total= 37 Term neonates	Neonatal physiology	Within 48 hours after birth	Plasma (UCB)	Hematopoietic stem/progenitor cells	To detect and characterize CD34^+^ microparticles in umbilical cord blood and analyze their associated microRNA profile.
Goetzl L. et al. (18)	2017	USA	Prospective cohort study	Total= 14 Term neonates with acute HIE	Neurological complications	At 8, 10, & 14 h after the initiation of therapeutic-controlled hypothermia	Serum (Peripheral blood)	Neurons	To evaluate whether neuronal exosome protein biomarkers from peripheral blood change over time after acute hypoxic brain injury and predict response to therapy in neonates with HIE.
Tan N. et al. (45)	2020	China	Prospective cohort study	Total N = 30 neonates, N = 20 with acute bilirubin encephalopathy, N = 10 Control neonates	Neurological complications	At diagnosis (Cases), Routine sampling (Controls)	CSF	Not determined	To identify differentially expressed proteins in microvesicles/exosomes isolated from the CSF of neonates with acute bilirubin encephalopathy, in order to help understand the pathogenesis of bilirubin-induced neurological injury and explore potential diagnostic biomarkers and therapeutic targets
Spaull R. et al. (40)	2018	United Kingdom	Observational case-series	Total= 3 preterm neonates with PHH	Neurological complications	Patient 1 & 2: Singletimepoint Patient 3: Day 25 after birth, Day 29, Day 32, Day 54, Day 124	CSF	Not determined	Isolate and characterise extracellular vesicles (exosomes) in the CSF of preterm infants with post-haemorrhagic hydrocephalus (PHH), and explore their potential as biomarkers of brain injury and disease progression.
Starke N. et al. (30)	2024	Germany	Pilot comparative study	Total= 15, N= 7 neonates on <30% FiO_2_, N= 7 neonates on >30% FiO_2_, N= 1 adult plasma sample (Control)	Neurological complications (Lung/brain axis)	Neonates: 1 week of age	Plasma (Peripheral blood)	Alveolar macrophages	To determine whether plasma EVs from preterm infants at risk for BPD contain increased alveolar macrophage–derived ASC and to investigate their role in mediating lung and brain injury via the lung–brain axis.
Jia R. et al. (47)	2015	China	Case-control study	Total= 20, N = 10 neonates (PE), N = 10 control neonates	Preeclampsia	At birth	Plasma (UCB)	Not determined	To identify and compare differentially expressed proteins in umbilical cord blood exosomes from normal and preeclamptic pregnancies and explore their potential role in the pathogenesis of preeclampsia.
Campello E. et al. (33)	2015	Italy	Case-control study	Total= 32 mother/infant pairs, N = 16 neonates from PE pregnancies, N = 16 neonates from non-PE pregnancies	Preeclampsia	At birth	Plasma (UCB-neonates & Peripheral blood-mothers)	Platelets Activated platelets Leukocytes Endothelial cells	To compare the levels and subtypes of circulating microparticles in maternal blood and venous umbilical cord blood between preeclamptic and normotensive pregnancies, in order to explore their association with preeclampsia.
Xueya Z. et al. (48)	2020	China	Experimental/Laboratory Observational study	Total= 38 mother/infant pairs, N = 18 neonates from PE pregnancies, N = 20 neonates from non-PE pregnancies	Preeclampsia	At birth	Plasma (UCB-neonate & Peripheral blood-mother)	Not recorded	To determine whether exosomal miR-125a-5p is dysregulated in preeclampsia and to explore its mechanistic role in trophoblast dysfunction via VEGFA modulation.
O'Reilly D. et al. (32)	2018	Ireland	Prospective cohort study	Total= 21 Preterm neonates (including 8 neonates with matched Day 1 and Day 3 samples)	Pregnancy compications, Extrauterine adaptation	Day 1 and/or day 3 post birth	Plasma (Peripheral blood)	Platelets	To determine whether the population and characteristics of circulating extracellular vesicles change in very preterm infants during the early postnatal transition period.
Simoncini S. et al. (29)	2017	France	Experimental/Laboratory Observational study	Total= 47, N = 29 preterm neonates, N = 18 term neonates	Pregnancy complications	At birth	UC-ECFCs	Endothelial cells	To investigate whether SIRT1 deficiency in ECFCs from premature neonates drives the biogenesis of pro-senescent EMPs, and to unravel the underlying epigenetic and signaling mechanisms.
Hujacova A. et al. (25)	2021	Czech Republic	Prospective cohort study	Total= 30, N = 20 preterm infants, N = 10 term infants	Hemostasis	At birth	Plasma (UCB)	Platelets, Activated platelets, Endothelial cells	To investigate the presence and levels of large platelet-derived and endothelial EVs in UCB of preterm newborns versus term controls, and to explore whether their levels correlate with hemolysis and gestational age, using conventional flow cytometry.
Kunte P. et al.	2023	India	Cross-sectional study	Total= 127 mother-infant pairs, N = 51 lean infants (lowest tertile of skinfold sum), N = 76 adipose infants (highest tertile of skinfold sum)	Pregnancy complications	At birth	Plasma (UCB)	Adipocytes	To examine whether adipocyte-derived EV microRNAs mediate maternal–fetal signaling, are associated with neonatal adiposity, and are influenced by maternal metabolic factors such as GDM, adiposity, and vitamin B12–folate status through their effects on adipogenic pathways.
Marell P. et al. (16)	2019	USA	Cross-sectional study	Total= 79 Term/near-term neonates (≥35 weeks) at risk of iron deficiency	Pregnancy complications	At birth	Plasma (UCB)	Neural	To determine whether cord blood-derived exosomal CNTN2 and BDNF can serve as non-invasive molecular markers of neonatal brain iron deficiency in infants exposed to maternal risk factors such as obesity, diabetes, or anemia.
Miranda J. et al. (37)	2018	Spain	Cross-sectional study	Total= 30, N = 10 IUGR, N = 10 SGA, N = 10 control	Pregnancy complications	At birth	Plasma (UCB)	Placental	To characterize and compare the profile of placenta-derived exosomes in maternal and fetal circulation in pregnancies complicated by FGR and SGA, compared with normal pregnancies.
Menon R. et al. (19)	2022	USA	Cross-sectional study	Total= 45 neonates, N = 13 preterm neonates, N = 10 neonates born after PPROM followed by preterm delivery, N = 10 term neonates, N = 12 term neonates born with elective cesarian section from pregnancies not in labor (TNIL)	Pregnancy complications	At birth	Plasma (UCB)	Not determined	To analyze and compare the proteomic cargo contents of exosomes isolated from umbilical cord blood plasma in term and preterm pregnancies, in order to understand fetal inflammatory signaling and its role in the initiation of labor.
Bruschi M. et al. (34)	2018	Italy	Experimental/Laboratory Observational study	Total= 6, N = 3 Term neonates, N = 3 Preterm neonates	Pregnancy complications	At birth	HUCMSCs culture supernatant	HUCMSCs	To compare the proteomic and metabolic profile of microvesicles released by umbilical cord mesenchymal stem cells from preterm versus term newborns, in order to establish a metabolic signature of prematurity.
Lal C.V. et al. (15)	2018	USA	Prospective cohort study	Total= 80 neonates, N = 18 ELBW neonates intubated at birth, N = 12 ELBW neonates for the validation of miRNA findings of the previous group, N = 25 Preterm neonates with established severe BPD at 36 weeks PMA, N = 25 Term or near-term neonates, intubated but without lung disease	Respiratory system	At birth (within 6 hours of life) At 36 weeks PMA	Tracheal aspirates	Airway epithelial cells Neutrophils	To identify airway exosomal miRNA signatures at birth that predict the development of severe BPD in extremely preterm infants, and to investigate the mechanistic role of miR-876-3p in BPD pathogenesis.
Ransom M.A. et al. (17)	2023	USA	Prospective cohort study	Total= 34 premature neonates	Respiratory system	Single timepoint (not determined)	Tracheal aspirates	Epithelial cells, Immune cells, Progenitor cells, Mesenchymal cells	To characterize EVs in tracheal aspirates of premature neonates across different stages of lung development, examining their size, composition, and surface markers, and to assess how EV profiles relate to gestational age and the risk of developing BPD.
Go H. et al. (52)	2020	Japan	Prospective cohort study	Total= 73 preterm infants (GA <32 w), N = 39 CLD group, N = 34, NCLD group	Respiratory system	At birth & at 28 DOL	Serum (UCB & Peripheral blood)	Not recorded	To evaluate microRNAs contained in serum EVs of premature infants as potential biomarkers for predicting the development of CLD.
Kanei S. et al. (51)	2025	Japan	Pilot comparative study	Total= 47, N = 33 neonates with ROP, N = 14 control neonates without ROP	ROP	At 34 weeks PMA	Tears	Not determined	To investigate whether miRNAs contained in tear-derived extracellular vesicles are associated with treatment-requiring retinopathy of prematurity and could serve as diagnostic biomarkers.

OD, Organ Dysfunction; NOD, Non-Organ Dysfunction; CHD, Congenital Heart Disease; CPB, Cardiopulmonary Bypass; ICU, Intensive Care Unit; ECMO, Extracorporeal Membrane Oxygenation; UCB, Umbilical Cord Blood; HDN, Hemolytic Disease of the Newborn; ABO-HDN, ABO Hemolytic Disease of the Newborn; Rh-HDN, Rhesus Hemolytic Disease of the Newborn; TF, Tissue Factor; TFPI, Tissue Factor Pathway Inhibitor; PDMP, Platelet-Derived Microparticles; VWF, Von Willebrand Factor; DOL, Day(s) of Life; NEC, Necrotizing Enterocolitis; HIE, Hypoxic-Ischemic Encephalopathy; CSF, Cerebrospinal Fluid; PHH, Post-Haemorrhagic Hydrocephalus; FiO_2_, Fraction of Inspired Oxygen; BPD, Bronchopulmonary Dysplasia; PE, Preeclampsia; IUGR, Intrauterine Growth Restriction; SGA, Small for Gestational Age; FGR, Fetal Growth Restriction; PPROM, Preterm Premature Rupture of Membranes; HUCMSCs, Human Umbilical Cord Mesenchymal Stem Cells; ELBW, Extremely Low Birth Weight; PMA, Postmenstrual Age; CLD, Chronic Lung Disease; ROP, Retinopathy of Prematurity; GA, Gestational Age; BW, Birth Weight.

**Figure 3:**
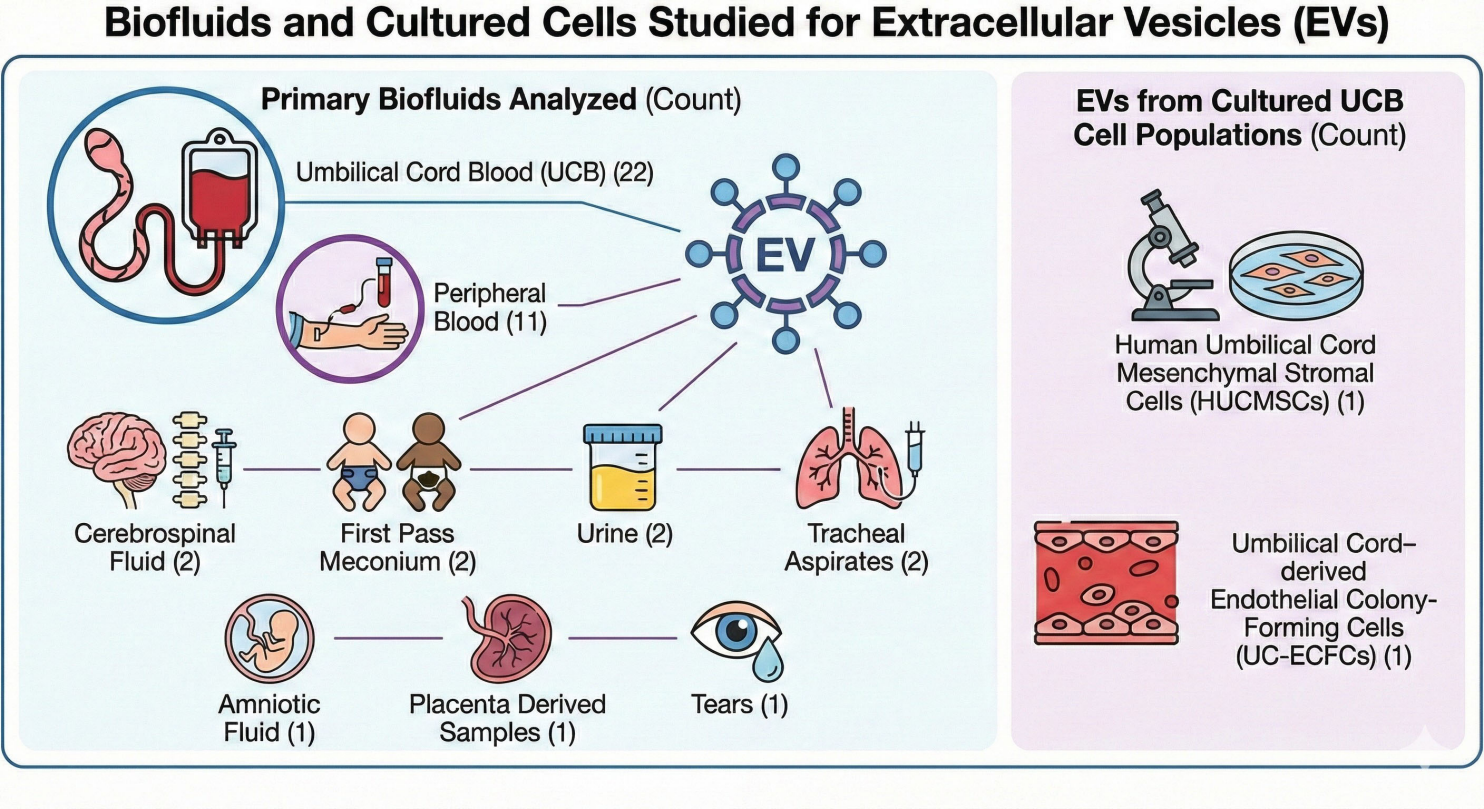
A summary of biofluids and cultured cell populations utilized for the study of extracellular vesicles, including the count of studies for each source. (Image created with Gemini based on data collected from the included studies and presented on **Tables 2**, **3**).

Sample collection most commonly was performed at birth or within the first 24–72 hours of life, although some studies included later postnatal time points during neonatal intensive care and disease related stage. Biological matrices analyzed across studies included umbilical cord blood (UCB) (22 studies), peripheral blood (11 studies), cerebrospinal fluid (2 studies), first pass meconium (2 studies), urine (2 studies), tracheal aspirates (2 studies), amniotic fluid (1study), placenta derived samples (1 study) and tears (1study). In addition, EVs were isolated from cultures of UCB cell populations such as human umbilical cord mesenchymal stromal cells (HUCMSCs) (1 study) and Umbilical Cord–derived Endothelial Colony-Forming Cells (UC-ECFCs) (1 study) (**Figure 3**).

#### 3.2.2 EV Size distribution, cellular origin and molecular cargo

EVs exhibit a high degree of structural and functional diversity, which is reflected in their varied physical dimensions, cellular sources, and bioactive content. Biogenesis-based subtypes include exosomes, derived from the endosomal multivesicular body pathway, and ectosomes (microvesicles or microparticles), which bud from the plasma membrane. However, these terms should be applied cautiously because most EV isolation methods are heterogeneous and definitive markers of biogenesis are lacking (3).

Across the included studies, EVs were predominantly characterized as heterogeneous populations encompassing microvesicles and exosome-sized particles, with reported size distributions most commonly ranging from approximately 100 nm to 1,000 nm, depending on the isolation and analytical method used. Smaller EVs, often classified as exosomes, were typically defined within the 30–150 nm range (15, 41, 48), whereas LEVs consistent with microvesicles extended up to 1 μm in diameter (11, 42, 49). With respect to cellular origin, EVs were most frequently derived from platelets (20, 22, 24, 25), endothelial cells (29, 33, 39) and leukocytes (33), but EVs with neuronal (18), adipocyte (43), airway epithelial cell origin (15) were also present as identified by surface expression of lineage-specific markers. At the molecular level, EV cargo included a range of surface antigens, adhesion molecules, and signaling molecules (proteins, miRNAs). Although the specific molecular profiles varied across studies, the overall qualitative pattern suggests that EV size, cellular origin, and cargo are closely aligned with underlying neonatal pathophysiological processes.

### 3.3 EVs in the perinatal period

EVs serve crucial biological processes aligned with their cellular origin. The functional capabilities of these tiny particles could reveal various aspects of the distinct neonatal physiology, especially in the critical period of extrauterine adaptation (**Table 2**).

**Table 2:** EVs in perinatal life.

Publication	Study population	Gestational age	Timepoint of sampling	Biofluid	Evs origin	EV Subtype and size	EV molecular signature	Main findings	EVs role
Xagorari A. et al. (31)	Total= 37 Term neonates	Not recorded	Within 48 hours after birth	Plasma (UCB)	Hematopoietic stem/progenitor cells	Microparticles (size: 100-300nm)	EV markers: CD34: hematopoietic stem/progenitor cell marker Annexin V: PS exposure EV cargo: miRNAs: miR-106b, miR-221, miR-517c, miR-519d, miR-520h	Human UCB contains a distinct population of CD34^+^ microparticles that can be reliably isolated and characterized, and which carry hematopoiesis-related microRNAs mirroring their parent stem cells.	Potential biomarkers of cord blood stem cell function.
Huang S. et al. (44)	Total= 10, N = 5 Term neonates, N = 5 adults	39.74w [± 0.70]	Neonates: At birth, Adults: single timepoint	Plasma (UCV & Peripheral blood)	Not determined	Exosomes (size: 40-150nm)	EV markers: CD63^+^/ALIX^+^: identity confirmation, EV cargo: 65 miRNAs differentially expressed, Upregulated in UCB: miR-122-5p, miR-127-3p, miR-1323, miR-192-5p, miR-487b-3p, miR-525-5p Downregulated in UCB: let-7b-5p, miR-10a-5p, miR-10b-5p, miR-125a-5p, miR-29a-3p. UCB-specific miRNAs: miR-219a-2-3p, miR-3157-5p, miR-369-3p, miR-4746-5p, miR-485-5p, miR-668-3p.	UCB and adult peripheral blood exosomes have similar size and concentration, but markedly different miRNA profiles, with 65 exosomal miRNAs differentially expressed (46 up- and 19 downregulated in UCB). These miRNAs mainly target pathways involved in pregnancy, cell migration, nervous system development.	Carriers of a pregnancy-specific miRNA signature that may regulate fetal development and biological processes distinct from adults.
Murphy C.A. et al. (11)	Total=167, N = 101 preterm, N = 66 term	Preterm: 28.3w [26.4 – 29.9] Term: 39.29w [38.9 – 39.9]	At birth (UCB) Day 1 (0–24 hours of life) Day 3 (Preterm: 48–72 h / Term: 25–96 h) Day 14–21 (preterm only)	Plasma (UCB & Peripheral blood)	Platelets, Endothelial cells Leukocyte cells	SEVs (size: <200nm) LEVs (size: 1000-1300nm)	EV markers: CD63, CD81: EV identity Annexin V: PS exposure CD41 (GPIIb), CD42b (GPIb), CD62P (P-selectin): Platelet origin VEGFR2: Endothelial origin, CD45: Leukocyte origin TF: procoagulant marker	Preterm birth triggers a distinct surge in circulating LEVs on the first 3 DOL, primarily platelet (CD42b+, CD62P+), endothelial (VEGFR2+), and TF–positive, accompanied by dynamic proteomic changes in EVs that reflect increased coagulation and inflammatory activity (↑F9, ↑APCS) and decreased vascular/structural proteins (↓VWF, ↓FN1, ↓ENO1), highlighting a unique postnatal adaptation profile not seen in term infants.	Biomarkers of platelet, endothelial and coagulation physiology during the first days of life.
O'Reilly D. et al. (32)	Total= 21 Preterm neonates (including 8 neonates with matched Day 1 and Day 3 samples)	Not recorded	Day 1 and/or day 3 post birth	Plasma (Peripheral blood)	Platelets	SEVs (size: 0–200nm) LEVs (size: 100–900nm) A distinct EV population of: 100–300nm on Day 3	Ev markers: CD41^+^ (GPIIb) Annexin V^+^: PS exposure	Major dynamic changes in plasma EVs, between 1 and 3 DOL in preterm neonates including: Increased EV concentration, Shift in EV size distribution, Altered EV composition, Decreased platelet-derived Evs	Biomarkers of platelet activation and EV composition during early postnatal life.
Ohta M. et al. (50)	Total= 20 preterm infants	34.1w [ ± 1.4]	At birth and at 2, 4, 8, 24, and 48 weeks of age.	Serum (Peripheral blood)	Not determined	Exosomes (size: not determined)	Not determined	Serum-derived exosome concentrations in preterm infants rise steadily through 48 weeks and are strongly influenced by GA at birth. GA—and to a lesser degree multiple birth—is associated with higher exosome levels, while respiratory disease shows no effect.	Biomarkers of physiological maturation and adaptation in preterm infants during early postnatal life.
Wasiluk A. et al. (36)	Total= 106, N = 51 preterm neonates, N = 55 term neonates	Preterm: 25–36w, Term: 38–41w	At birth	Plasma (UCB)	Platelets	Microparticles (size: not measured)	CD61 (GPIIb/IIIa complex)	Preterm newborns, present higher percentages of platelet-derived MPs suggesting a compensatory mechanism for the immature hemostatic system. These differences are independent of platelet count and are more pronounced in preterm males than females.	Potential biomarkers of platelet activation and a potential compensatory component of hemostasis in preterm newborns.
Keller S. et al. (41)	Total= 20 subjects, N = 5 newborn urine samples, N = 11 adult urine samples, N = 4 amniotic samples from women undergoing amniocentisis	Not recorded	Newborn urine: postnatally (not specified), Amniotic sampling: 14-18w	Urine	Kidneys	Exosomes (size: 20-100nm)	CD24: urinary and amniotic fluid exosome marker CD9: tetraspanin exosome marker HSP70: heat shock protein Annexin-I: exosomal membrane protein, Aquaporin-2: renal tubular origin	CD24^+^ exosomes are present in both newborn urine and in amniotic fluid as early as 14–18 weeks GA, and are derived from fetal kidney cells rather than the mother. Exosomes may play a role in fetal–maternal interaction & developmental kidney biology.	Potential mediators of fetomaternal communication.
Turunen J. et al. (2021) (27)	Total= 44 Term neonates, N = 23 neonates (vaginal deliveries), N = 21 neonates (C-sections)	39.4 [ ± 1.8]	Meconium: within first 24h of life, Placenta: Immediately after delivery. Amniotic fluid: at delivery	First pass Meconium, Amniotic fluid, Placenta	Not determined (possibly bacterial)	Microvesicles (size: 100 ± 200nm)	Not determined	First-pass meconium contains abundant EVs and a detectable microbiome, especially in vaginally delivered infants. Placenta and amniotic fluid show minimal bacterial presence. EVs may mediate maternal-fetal transfer of microbial DNA or signals, potentially contributing to *in utero* gut colonization.	Potential vehicles for maternal–fetal microbial material transfer contributing to early gut colonization.
Turunen J. et al. (28)	Total= 17 Term neonates, N = 5 neonates (C-section), N = 6 neonates (vaginal delivery without intrapartum antibiotics), N = 6 neonates v(aginal delivery with intrapartum antibiotic exposure)	39.4 [ ± 1.4]	Within first 24h of life	First pass Meconium	Gram+ & Gram- Bacteria	Exosomes (small EVs) (Size not determined)	EV cargo: Bacterial 16S rRNA (V4–V5 variable region amplified) RNA from phyla: Firmicutes (62%) Actinobacteriota (18%) Proteobacteria (10%) Bacteroidota (7.3%) Most abundant genera: Streptococcus (21%), Staphylococcus (17%), Anaerococcus (12%), Corynebacterium (10%)	Bacterial EVs containing bacterial RNA were identified in all first-pass meconium samples. The EV-associated bacterial communities were dominated by Firmicutes and Staphylococcus/Streptococcus genera, and were not significantly different by delivery mode or intrapartum antibiotic exposure.	Novel microbiome components
Wang D. J. et al. (46)	Total= 70 UCB samples from term neonates	38.51w [± 1.67]	At birth	UCB	Not derermined	Microvesicles (size: 167 ± 77.1 nm)	EV markers: CD63: identity confirmation, EV cargo: 337 miRNAs identified, 85 lactation-related.	Human UCB–derived microvesicles contain numerous lactation-related microRNAs and are functionally active. They can be taken up by mammary epithelial cells and significantly increase β-casein secretion *in vitro*, suggesting a role in feto-maternal signaling related to lactation.	Mediators of fetal–maternal signaling in lactatogenesis.

UCB, Umbilical Cord Blood; UCV, Umbilical Cord Vein; EVs, Extracellular Vesicles; SEVs, Small Extracellular Vesicles; LEVs, Large Extracellular Vesicles; PS, Phosphatidylserine; TF, Tissue Factor; GA, Gestational Age; DOL, Day(s) of Life; GPIIb, Glycoprotein IIb; GPIb, Glycoprotein Ib; VEGFR2, Vascular Endothelial Growth Factor Receptor 2; miRNA, microRNA; FN1, Fibronectin 1; VWF, Von Willebrand Factor; ENO1, Enolase 1; APCS, Amyloid P Component Serum; CD, Cluster of Differentiation.

Studies demonstrated that UCB from healthy term neonates contains a distinct population of CD34^+^-derived MPs that can be reliably isolated and exhibit miRNA expression patterns closely matching those of their parental hematopoietic stem cells (HSCs) (31). These MPs are enriched in hematopoiesis-related miRNAs involved in cell cycle regulation, self-renewal, erythroid differentiation, and cell–cell adhesion, including members of the miR-106 and miR-221 families and the chromosome 19q13 cluster. The strong concordance between miRNA profiles of CD34^+^ cells and their corresponding MPs indicates that stem cell–derived MPs preserve the molecular fingerprint of their cells of origin, highlighting their potential value as noninvasive biomarkers in UCB (31).

Additional analyses showed that UCB-derived exosomes have particle sizes and concentrations comparable to those found in adults, yet display distinct miRNA signatures. High-throughput sequencing identified 65 differentially expressed exosomal miRNAs in UCB, including 46 upregulated and 19 downregulated species (44). Functional enrichment using Gene Ontology and KEGG (Kyoto Encyclopedia of Genes and Genomes) pathways revealed preferential involvement in pathways related to pregnancy and reproduction, cell migration, nervous system development, and exosome biogenesis, including mTOR (mammalian target of rapamycin), MAPK (mitogen-activated protein kinase), insulin, and neurotrophin signaling. Several miRNAs uniquely enriched in UCB (miR-125a-5p, miR-302a-5p, miR-126-5p, and miR-223-3p) were associated with embryonic development, stem cell differentiation, immune regulation, and tumor suppression, supporting the presence of a pregnancy-specific exosomal signature and a specialized regulatory role of UCB-derived exosomal miRNAs in fetal growth and developmental processes (44).

Serum-derived exosome levels in preterm infants increase progressively up to 48 weeks and are primarily determined by GA at birth. Higher GA—and to a lesser extent multiple gestation—was associated with elevated exosome concentrations, whereas respiratory disease presented no significant impact (50). Given the role of exosomes in promoting immune and gastrointestinal maturation, infants born at earlier GA are likely to exhibit substantially lower exosome levels both at birth and during the postnatal period (54, 55). GA and multiple birth independently predicted higher exosome levels, even in the absence of clinically apparent differences between twins and singletons (50). It is suggested that perinatal factors more commonly seen in multiple pregnancies may influence exosome production, possibly through alterations in the intrauterine environment, placental dynamics, or pregnancy-related stressors (56). These changes may promote increased exosome release as an adaptive or compensatory biological response to multiple cellular interactions rather than reflecting overt pathology. However, the underlying mechanisms were not directly examined and require further investigation.

In the study of Murphy et. al (11), preterm birth was associated with rapid and dynamic alterations in circulating EVs during early adaptation to extrauterine life. Marked increases in both SEV and LEV concentrations were observed between Day 1 and Day 3 of life in preterm infants, accompanied by shifts in vesicle size, cellular origin, and molecular cargo. By day 3 of life, the increased EV population in preterm neonates predominantly derived from platelets, endothelial cells, and TF^+^ EVs. These findings indicate enhanced platelet activation, endothelial involvement, and vascular signaling during early postnatal adaptation in preterm infants. Proteomic profiling revealed distinct temporal signatures involving proteins linked to haemostasis, pulmonary physiology, inflammation, and innate immunity, indicating that EVs likely participate in key biological processes during neonatal transition (11). These changes were substantially greater than those observed in healthy term infants, highlighting a strong influence of prematurity rather than normal postnatal maturation alone. It seems that timing of sample collection is critical as EV profiles change rapidly in the early neonatal period and may confound disease interpretation if not standardized. Additionally, UCB is a feasible EV source, but it does not fully represent postnatal EV biology and should be interpreted cautiously.

O’Reilly et al. in a sub-cohort including preterm neonates, reported that extrauterine transition period was associated with a significant increase in circulating EVs between Day 1 and Day 3 of life, involving both SEVs and LEVs. This increase was accompanied by shifts in EV size distribution, composition, and protein expression, including a relative reduction in PDEVs. Collectively, these findings support the concept that exaggerated EV release and dynamic cargo remodeling in preterm infants reflect developmental hemostatic adaptation and potential endothelial perturbation, a finding of particular relevance given the growing evidence linking prematurity with later endothelial dysfunction and cardiovascular risk (57–59). In addition platelets and their activation are crucial in the ductus arteriosus closure. Platelets may play a regulatory role in ductal closure beyond luminal occlusion, potentially mediating tissue remodeling through PDEV–driven cell-to-cell communication (60), a mechanism that warrants further investigation in preterm infants.

UCB blood showed a procoagulant profile that is potentially attributed to relevant procoagulant drivers. Placental tissues, including trophoblasts, amniotic fluid, and myometrium, constitutively express high levels of TF as part of the “obstetrical hemostatic envelope” (61, 62), a mechanism that protects against hemorrhage and may be further amplified by labor-associated oxidative stress or tissue injury during cord handling (63). Placental blood contains increased numbers of TF-bearing EVs, released from activated endothelial cells and trophoblasts under conditions of inflammation or hypoxia, providing a potent surface for coagulation activation and contributing to the observed procoagulant imbalance (35).

Furthermore, accumulating evidence indicates that EVs are key mediators of fetal-maternal crosstalk during the perinatal period. Keller et al. (41) demonstrated the presence of CD24-positive exosomes in neonatal and adult urine as well as in mid-gestation amniotic fluid, indicating early and sustained exosomal secretion and supporting a role for EV in fetal–maternal communication. Experimental and genetic models confirmed the fetal, kidney-derived origin of these exosomes and showed that CD24 functions as a marker rather than an essential component of exosome biogenesis.

In 2021, Turunen et al. (27) assessed the concept of fetal gut colonization in placental, amniotic and first-pass meconium samples. First-pass meconium contained abundant EVs (microvesicles) and a distinct, delivery mode–dependent microbiome, with higher bacterial diversity and load observed in vaginally delivered infants. In contrast, placental and amniotic fluid samples displayed minimal bacterial signals. The presence of abundant EVs in meconium suggests a potential role for vesicle-mediated maternal–fetal transfer of microbial DNA or bioactive signals, which may contribute to early gut microbial exposure and *in utero* immune priming. A second study of Turunen et al. (28) in 2023 exhibited the presence of bacterial EVs in all first-pass meconium samples, with dominant taxa belonging to Firmicutes, Actinobacteriota, Proteobacteria, and Bacteroidota, and Streptococcus and Staphylococcus as the most abundant genera. The taxonomic composition and diversity of bacterial EVs did not differ significantly by delivery mode or intrapartum antibiotic exposure, suggesting that EV-associated microbial signals are a consistent feature of the early gut environment. Together, these studies suggest that EVs may represent a key mechanism by which microbial signals reach the fetal and neonatal gut during intrauterine and early perinatal life. The potential ability of bacterial EVs to cross biological barriers, could enable host–microbe interactions to occur even in the absence of established whole-cell bacterial colonization.

In the study of Wang et al (46). al microvesicles isolated from human UCB expressed CD63 and carried a broad spectrum of miRNAs, including 85 lactation-related miRNAs identified through bioinformatic and expression analyses. *In vitro*, these microvesicles were rapidly taken up by human mammary epithelial cells and significantly increased β-casein secretion, supporting the EV-mediated fetal–maternal communication in the regulation of lactation. Overall it becomes apparent that EVs emerge as central carriers of biological information at the maternal–fetal interface, integrating developmental, immunological, and metabolic signaling during intrauterine and early perinatal life.

EVs play a central role as carriers of biological information and mediators of intercellular signaling in both the intrauterine and neonatal environments. They provide critical insight into pregnancy pathophysiology, fetal-maternal communication, fetal development, neonatal outcomes, and mother–infant interactions, including the establishment of lactation (11, 33, 46). EV profiles reflect fetal and neonatal physiology, the dynamic maturation of immune and haemostatic systems, and endothelial function, supporting the concept of preterm birth as a developmental condition associated with an early biological imprint of accelerated maturation (11). Collectively, these findings establish a comprehensive framework for understanding the functional significance of EVs during the transition from intrauterine to extrauterine life and early neonatal development.

### 3.4 EV role in various neonatal clinical contexts

Gathering the current literature on EVs assessed in the neonatal population, provides a clear view of their subtle but universal role in neonatal health and disease.

Research spans fetal and neonatal physiology as well as extrauterine adaptation of term and preterm infants where characterization of circulating EVs and their molecular cargo reflect transitional and developmental changes after birth (11, 31, 41, 44, 50). Within maternal–fetal medicine, EVs were assessed in multiple pregnancy complications such as preeclampsia (PE) (33, 47, 48), or FGR (37) and as mediators of maternal–infant communication affecting neonatal adaptation, adiposity and lactation (16, 19, 25, 29, 32, 34, 43, 46). Emerging areas also include the role of EVs in the microbiome of first pass meconium, where they present as novel components, of host-microbiome interactions capable of crossing biological barriers and delivering microbial signals without the presence of whole-cell bacteria (27, 28). Furthermore, EVs were extensively studied in neonatal hemostasis, with PDEVs and endothelial EVs evaluated as key contributors to hemostatic physiology and related disorders (9, 20, 22–24, 26, 35, 36, 38, 53). In hemolytic disease of the newborn (HDN) and hyperbilirubinemia, particularly endothelial and erythrocyte-related EVs, revealed further implications of HDN and were linked to hemolysis, and disease severity (39, 42, 49). In CHD requiring cardiac surgery, EV-derived miRNAs and circulating EV profiles were explored in relation to postoperative recovery (21), while in critically ill neonates, including those on extracorporeal membrane oxygenation (ECMO), endothelial‐derived EVs were assessed as indicators of vascular injury (8). Additional work examines EV involvement in NEC (14), ROP (51), respiratory disease (15, 17, 52), and neurological complications including brain–lung axis interactions (18, 30, 40, 45). Collectively, these studies demonstrate that EVs serve diverse roles and are explored across nearly all major neonatal and perinatal clinical domains (**Table 3**).

**Table 3:** The role of EVs in various clinical fields.

Publication	Clinical Field	Study population	Gestational age (weeks)	Timepoint of sampling	Biofluid	Evs origin	EV Subtype and size	EV molecular signature	Main findings	EVs role
Alhamdan F. et al. (21)	CHD / Postoperative outcomes	Total = 10 neonates, N = 5 neonates with OD, N = 5 neonates with NOD	Not recorded	T0 – Before surgery begins T1 – During the rewarming phase of CPB T2 –Upon admission to the ICU after surgery T3 – On postoperative day 1	Plasma (Peripheral blood)	Not recorded	Exosomes (Size: 131.6nm OD/131.9nm NOD)	EV markers: ALIX, TSG101 CD63, CD81, EpCAM Annexin A5, Flotillin-1, ICAM1, GM130: cellular contamination marker, EV cargo: miR-200b-5p, miR-4800-5p, miR-363-3p, miR-483-5p	Infants with postoperative OD showed distinct EV-miRNA patterns marked by persistent apoptosis, inflammation, NET activation, and complement consumption, especially toward the end of surgery.	Prognostic biomarkers Pathophysiological mediators
Galley J.D. et al. (14)	NEC	Total= 42, N = 9 medical NEC cases, N = 13 surgical NEC cases, N = 8 non-NEC sepsis cases, N = 12 Controls	≤ 34 weeks gestational age	T1: Within 24 hours of enrollment (immediately after diagnosis) T2: 2 to 5 days after enrollment (during disease) T3: 6 to 14 days after enrollment (recovery phase)	Urine	Not determined	EVs (Size: 100-200nm)	EV markers: Alix, CD63 (Identity), EV cargo miRNA cargo (from sequencing; enriched for miRNAs) 26.08% mapped miRNAs. Many differentially expressed miRNAs in NEC vs controls/sepsis, including: miR-376a-3p, miR-518a-3p, miR-604, miR-139-3p, miR-31-5p, etc.	Urine-derived EVs from premature infants contain abundant miRNAs, several of which are significantly dysregulated in NEC compared to sepsis and healthy controls. These EV-miRNA signatures correlate with inflammatory and injury-related molecular pathways, supporting their potential as non-invasive NEC biomarkers.	Non-invasive diagnostic biomarkers reflecting NEC-specific inflammatory molecular activity.
Vítková V. et al. (8)	Critically ill neonates	Total= 26, N = 13 neonates on ECMO, N = 14 healthy neonates	ECMO: 38.9 [± 0.4], Control: 39.4 [± 0.3]	Controls: 48–72 h of life ECMO group: 36–350 h after ECMO initiation	Plasma (Peripheral blood)	Endothelial cells Mucosal cells	Microvesicles (Size: not derermined)	Annexin V: PS exposure CD31 (PECAM-1):endothelial/platelet marker CD105 (Endoglin): endothelial activation marker VEGFR2 (CD309): endothelial angiogenic receptor MadCAM-1: mucosal endothelial marker	Critically ill newborns on ECMO had significantly elevated levels of endothelial MVs, particularly Annexin V^+^/MadCAM-1^+^ MVs, compared with healthy controls, indicating pronounced endothelial activation and injury. Elevated microvesicles also correlated with higher inflammatory markers and worse clinical severity.	Biomarkers of endothelial injury and vascular dysfunction
Awad H.A. et al. (42)	HDN	Total= 85 neonates, N = 45 ABO-HDN, N = 20 Rh-HDN, N = 20 Control	ABO HDN: 37.8 [± 1.1], Rh HDN: 37.9 [± 1.1], Control: 38 [± 1.4]	Pre and post exchange transfusion and/or phototherapy	Plasma (Peripheral blood)	Endothelial cells	Microparticles (Size: 200 nm to 2000 nm)	CD144 (VE-cadherin): primary endothelial marker	Neonates with ABO HDN had significantly higher circulating CD144^+^ EMPs than both Rh HDN neonates and healthy controls, with the highest levels seen in those with severe hyperbilirubinemia. EMP levels decreased significantly, especially following exchange transfusion, and correlated with markers of hemolysis, supporting a link between ABO incompatibility and endothelial injury.	Biomarkers of endothelial injury and disease severity
Zhu X.J. et al.	HDN	Total= 72 neonates, N = 29 ABO-HDN, N = 22 Rh-HDN, N = 21 Controls	ABO HDN: 38.1 (± 1.2) Rh HDN: 39 (± 1.1) Controls: 40 (± 1.5)	Pre and post exchange transfusion and/or phototherapy	Plasma (Peripheral blood)	Endothelial cells	Microparticles (Size: 200 nm to 2000 nm)	CD144 (VE-cadherin): primary endothelial marker	Neonates with ABO and Rh HDN showed significantly higher pre-therapy CD144^+^ EMP levels and von vWF Ag compared with healthy controls, with the highest levels in the ABO HDN group. EMP levels decreased significantly after exchange transfusion and/or phototherapy and correlated with hemoglobin, LDH, and indirect bilirubin levels, linking EMPs to disease severity and endothelial dysfunction.	Biomarkers of endothelial injury and disease severity
Yücesoy E. et al. (39)	Hyperbilirubinemia	Total = 58, N = 33 Non hemolytic hyperbilirubinemia cases, N = 25 Controls	Cases: 38.86 [± 1.10] Controls: 39.69 [± 1.18]	Pre and post phototherapy (Cases), Single timepoint (Controls)	Plasma (Peripheral blood)	Endothelial cells, Platelets	Microparticles (Size: <1.5 µm)	Annexin V^+^: Total apoptotic MPs Annexin V^+^/ CD31^+^ CD144^−^: Platelet-derived apoptotic MPs: Annexin V^+^ / CD144^+^Endothelial-derived apoptotic MPs Annexin V^+^ / CD142: TF^+^ apoptotic MPs	Neonates with nonhemolytic pathological hyperbilirubinemia had significantly higher total apoptotic and endothelial-derived apoptotic MP levels compared with healthy controls, both before and after phototherapy. Phototherapy significantly reduced bilirubin levels but did not significantly change apoptotic MP levels, suggesting persistent bilirubin-related endothelial and cellular apoptosis.	Diagnostic biomarkers of bilirubin-induced endothelial and cellular injury
Hujacova A. et al.	Hemostasis	Total= 30, N = 20 preterm infants, N = 10 term infants	Preterm: 31.5w [± 2], Term: 38.1 [ ± 1.8]	At birth	Plasma (UCB)	Platelets, Activated platelets, Endothelial cells	Microparticles (size: 150-300nm)	CD41^+^ CD36^+^: Platelet MPs CD41^+^ CD62P^+^: Activated platelet MPs CD31^+^ CD105^+^: Endothelial MPs	Large platelet, activated platelet, and endothelial EVs in UCB were similar between preterm and term infants, with no GA–dependent differences, although CD31 signal intensity was lower in preterm neonates.	Potential biomarkers reflecting platelet and endothelial status in preterm versus term newborns.
Hujacova A. et al.	Hemostasis	Total= 12, N = 5 preterm infants, 7 term infants	Term: 39 [± 0.6], Preterm: 31.6 [± 2.7]	At birth	Plasma (UCB)	Platelets	LEVs (Size: 500–900 nm)	CD36^+^ (thrombospondin receptor)/ CD41^+^(GPIIb) CD41^+^ (GPIIb)/ CD62^+^(P-selectin)	Preterm and term newborns had similar UCB PEV counts. Preterm PEVs showed lower CD36 and CD62 fluorescence which likely reflects reduced platelet activation or immaturity in preterm newborns.	Biomarkers of platelet maturation, Biomarkers of sample handling effects
Uszynski M. et al. (9)	Hemostasis	Total= 56, N = 28 mother-infant pairs	Term: 39.3 [38.0–41.6]	At bith	Plasma (UCB)	Not determined	Microparticles (Size: not determined)	Annexin V: PS exposure, TF & TFPI: expressed on the surface of microparticles	UCB contained significantly higher levels of procoagulant MPs compared with maternal blood, alongside higher TF antigen and lower TFPI antigen levels, suggesting that MPs contribute to maintaining effective thrombin generation despite the immaturity of the fetal coagulation system. MPs may act as compensatory procoagulant elements in neonatal hemostasis.	Pathophysiological mediators which may compensate for the immaturity of the fetal hemostatic system
Korbal P. et al. (35)	Hemostasis	Total= 48, N = 23 preterm infants, 25 term infants	Preterm: median 34 (IQR 32–36), Term: median 40 (IQR 39–41)	At birth	Plasma (UCB)	Not determined	Microparticles (size: not determined	TF: expressed on the surface of microparticles	TF–bearing MPs showed higher levels in preterm infants compared with term infants, although the difference was marginally significant. When both groups were combined, MPs-TF levels correlated negatively with gestational age and birth weight, suggesting an association between prematurity and increased procoagulant microparticle burden.	Biomarkers of prothrombotic risk in preterm newborns.
Wasiluk A. et al. (36)	Hemostasis	Total= 106, N = 51 preterm neonates, N = 55 term neonates	Preterm: 25–36 Term: 38-41	At birth	Plasma (UCB)	Platelets	Microparticles (Size: not determined)	CD61 (GPIIIa / GPIIb-IIIa complex)	Preterm newborns had a significantly higher proportion of PDMP compared with term newborns, with male preterm infants showing the highest PDMP levels. The authors suggest that increased PDMP formation in preterms may serve as a compensatory mechanism for immature neonatal hemostasis.	Pathophysiological mediators which may compensate for the immaturity of the neonatal hemostatic system
Schweintzger S. et al. (23)	Hemostasis	Total= 59, N = 31 Term neonates, N = 28 adults	Term: 38-40	Neonates: at birth, Adults: single timepoint	Plasma (UCB)	Not determined	Microparticles (Size: <1μm)	Annexin V^+^: PS exposure	MP numbers were not significantly increased in newborn cord blood compared with adults, but their procoagulant activity was significantly higher, as shown by ELISA and thrombin generation assays. This suggests that neonatal MPs have a greater functional impact on hemostasis.	Pathophysiological/ functional mediators of neonatal hemostasis
Schweintzger S. et al. (24)	Hemostasis	Total= 59, N = 31Term neonates, N = 28 adults	Term neonates, GA Not specified	At bitrh	Plasma (UCB & Peripheral blood)	Platelets	Microparticles (Size: <1μm)	Annexin V^+^: PS exposure	UCB showed a slightly higher though not significant total MP concentration compared with adults, whereas MP‐dependent procoagulant activity was significantly higher in newborns. This suggests that neonatal MPs, although similar in number, have a greater functional contribution to thrombin generation and neonatal hemostasis.	Pathophysiological/ functional mediators of neonatal hemostasis
Peñas-Martínez J. et al. (38)	Hemostasis	Total= 26, N = 14 Term neonates, N = 12 adults	Term: 38-41	Neonates: at birth, Adults: single timepoint	Plasma (UCB & Peripheral blood)	Not determined	Exosomes (Size: neonates: 30–50 nm, adults: 50–80 nm & 80–120 nm)	EV markers: TSG101: exosome marker CD41 (GPIIb) CD63: exosome tetraspanin marker EV cargo: 131 differentially expressed proteins between neonates & adultes.Overexpressed in neonatal exosomes: Integrins αIIb, β3 GNAI2, TLN1, RAP1A, CD9, CD36 PF4, vWF, FV, FVIII, fibrinogen (α, β, γ), A2MG, Underexpressed in neonatal exosomes: mainly immunoglobulins and protein S	Neonatal exosomes are significantly smaller and show a procoagulant-enriched proteomic profile, with increased expression of platelet activation and clotting cascade proteins. These age-dependent differences suggest a developmentally adapted hemostatic profile that may contribute to transfusion-related mismatch risks when adults platelet products are given to newborns.	Pathophysiological & developmental mediators of hemostatic balance.
Karlaftis V. et al. (53)	Hemostasis	Total =120, N = 10 term neonates, N = 80 children, N = 20 adults	Term: >37	Neonates: 1 & 3 DOL, Children & Adults: Single timepoint	Plasma (Peripheral blood)	Not determined	Microparticles (Size: not determined)	No specific markers analyzed	Healthy neonates (day 1 and day 3) displayed significantly longer PPL clotting times, indicating lower MP procoagulant phospholipid activity compared with other age groups. This suggests functionally fewer procoagulant MPs, highlighting developmental differences in microparticle-related coagulation activity in neonates.	Pathophysiological mediators of age-dependent differences in plasma procoagulant activity
Schmugge M. et al. (22)	Hemostasis	Total= 94, N = 38 Term neonates with UCB samples, N = 19 neonates with peripheral blood samples 2–3 DOL (15 of which were paired repeat samples from the UCB group, 4 were sampled only once), N = 15 Children, N = 22 Adults	Term: 39.3	Neonates: At birth, 2–3 DOL Children & Adults: Signle timepoints	Plasma (UCB & Peripheral blood)	Platelets	Microparticles (Size: <0.8μm)	CD41 (GPIIb) Annexin V: PS exposure	Platelets from healthy neonates (especially at 2–3 DOL) showed higher baseline activation and increased MP formation compared to adults, with a significant correlation between VWF binding and platelet activation markers, including annexin V binding. Neonatal platelets are potentially more prone to activation and MP release in the early postnatal period, possibly due to increased large VWF multimers.	Biomarkers of enhanced PLT activation and procoagulant surface exposure in neonates
Michelson A.D. et al. (20)	Hemostasis	Total=28, N = 7 Term neonates, N = 8 Preterm neonates, N = 13 adults	Term: 38-41 Preterm: 24-30	Neonates: at birth, Adults: single timepoint	Plasma (UCB & Peripheral blood)	Platelets	Microparticles (Size: <770nm)	GPIb (CD42b) Factor V/Va binding sites: surface marker of procoagulant activity	Preterm neonates generated a higher percentage of PDMPs than adults and term neonates, but their PDMPs and platelets showed markedly reduced Factor V/Va binding and procoagulant activity, due to a relative deficiency of plasma Factor V. This functional defect was corrected by adding adult plasma or exogenous Factor V, suggesting a mechanism contributing to the increased risk of IVH in preterm infants.	Pathophysiological mediators of impaired neonatal procoagulant capacity and potential IVH risk in preterm infants.
Spaull R. et al. (40)	Neurological complications	Total= 3 preterm neonates with PHH	Preterm: 23-24	Patient 1 & 2: Singletimepoint Patient 3: Day 25 after birth, Day 29, Day 32, Day 54, Day 124	CSF	Not determined	Patient 1: 42.5 nm Patient 2: 42.5 nm Patient 3: 32.5 nm	EV surface markers:CD63^+^, CD81^+^ EV cargo: miR-9, miR-17, miR-26a, miR-124, miR-1911	Exosomes are present in the CSF of preterm infants with PHH and carry brain-related microRNAs. Exosome concentration decreased over time following PHH, while miR-1991 increased suggesting dynamic changes in EV profiles following PHH	Pathophysiological and potential prognostic biomarkers of brain injury
Tan N. et al. (45)	Neurological complications	Total N = 30 neonates, N = 20 with acute bilirubin encephalopathy, N = 10 Control neonates	Moderate ABE: 38.60 [ ± 1.40], Severe ABE: [38.20 ± 1.70], Control: 38.40 [ ± 1.34]	At diagnosis (Cases), Routine sampling (Controls)	CSF	Not determined	Exosomes (Size: neonates: 75–200 nm, peak: 103nm)	EV markers: CD9, EV cargo: 291 Differentially expressed Key ABE-associated EV proteins: Upregulated: LTF (lactoferrin), DEFA1 (A-defensin 1), immunoglobulin domains, complement C4B/C5 Downregulated: S100A7, S100A9, Functional enrichment: NF-κB, PI3K-AKT, MAPK.	Patients with acute bilirubin encephalopathy had distinct proteomic alterations in CSF-derived microvesicles/exosomes, with 291 differentially expressed proteins, primarily linked to immune-inflammatory and neuroinflammatory pathways, reflecting active molecular processes underlying bilirubin-induced brain injury.	Pathophysiological and diagnostic biomarkers of bilirubin-induced neuroinflammation and brain injury
Goetzl L. et al. (18)	Neurological complications	Total= 14 Term neonates with acute HIE	Term: 38-41	At 8, 10, & 14 h after the initiation of therapeutic-controlled hypothermia	Serum (Peripheral blood)	Neuronal	Exosomes (134 nm ± 46.6 nm)	EV markers: Contactin-2 / TAG1 (neuronal membrane adhesion protein), EV cargo: Synaptopodin, Synaptophysin, Neuron-Specific Enolase, Cytochrome c oxidase subunit IV	The slope of change in exosomal synaptopodin between 8 and 14 hours of therapeutic hypothermia strongly predicted clinical outcomes in neonatal HIE, including length of stay, discharge on anticonvulsants, and abnormal neuroimaging scores.	Potential diagnostic and prognostic biomarkers of HIE and treatment response.
Marell P. et al. (16)	Neurological complications	Total= 79 Term/near-term neonates (≥35 weeks) at risk of iron deficiency	Male: 39.6 [ ± 1.2] Female: 39.4 [ ± 1.2]	At birth	Plasma (UCB)	Neuronal	Exosomes (Size: not determined)	EV markers: CD81, CNTN2 ( neuronal-specific exosomal marker), EV cargo: CNTN2: neural adhesion protein BDNF (Brain-Derived Neurotrophic Factor)	UCB exosomal CNTN2 positively correlated with ferritin, suggesting an association with neonatal brain iron status, while exosomal BDNF levels inversely correlated with ferritin, particularly in females. Maternal diabetes showed sex-specific effects, with CNTN2 increasing in females but decreasing in males.	Biomarkers of of neonatal brain iron status
Starke N. et al. (30)	Neurological complications (Lung/brain axis)	Total= 15, N= 7 neonates on <30% FiO_2_, N= 7 neonates on >30% FiO_2_, N= 1 adult plasma sample (Control)	LO2: 27.8w [± 1.3], HO2: 24.3w [± 0.5],	Neonates: 1 week of age	Plasma (Peripheral blood)	Alveolar macrophages	Exosomes (Size: 10-150nm)	EV markers: CD63 (tetraspanin), ASC (apoptosis), Caspase-1 (inferred from inflammasome signaling context) IL-1β pathway signaling associations (systemic inflammation)	ASC^+^ EVs, likely originating from early pulmonary or systemic inflammasome activation, were significantly elevated in infants who developed IVH, supporting ASC^+^ EVs as mediators of the lung–brain inflammatory axis in preterm brain injury.	Biomarkers of preterm brain injury risk & Pathophysiological mediators of early inflammasome activation contributing to preterm brain injury.
Campello E. et al. (33)	Preeclampsia	Total= 32 mother/infant pairs, N = 16 neonates from PE pregnancies, N = 16 neonates from non-PE pregnancies	Preeclampsia group: 38w [± 6 d] Normotensive group: 37w [± 1 d]	At birth	Plasma (UCB & Peripheral blood)	Platelets Activated platelets Leukocytes Endothelial cells	Microparticles (Size: 0.1-1μm)	EV markers: Annexin V-FITC (defines total MPs), CD61-PE (platelet MPs), CD62P-PE (P-selectin) (activated platelet MPs), CD62E-PC5 (endothelial MPs), CD45-PC5 (leukocyte MPs), CD142-PE (TF) (TF-bearing MPs)	Preeclampsia correlated with significantly higher total MPs, activated platelet MPs, leukocyte MPs, and TF-bearing MPs in both maternal and cord blood, while platelet-derived MPs (CD61^+^) were lower in PE. Cord blood had consistently higher MP levels than maternal blood, suggesting a hypercoagulable fetal environment in PE.	Pathophysiological mediators: procoagulant and pro-inflammatory mediators contributing to the vascular dysfunction of preeclampsia.
Jia R. et al. (47)	Preeclampsia	Total= 20, N = 10 neonates (PE), N = 10 control neonates	Controls: 38.6 [ ± 5.5] Cases: 34.3 [ ± 4.6]	At birth	Plasma (UCB)	Not determined	Exosomes: (Size: PE: 120 ± 37 nm, Control: 112 ± 40 nm)	EV cargo: Proteome (LC–MS/MS): 29 differentially expressed proteins between PE vs control: 14 upregulated, 15 downregulated Key dysregulated pathways: Complement & coagulation cascades Enzyme regulator activity Extracellular region proteins Nine proteins central to complement/coagulation: C4BPA, C4BPB, F13B, FGA, FGB, FGG, MBL2, PROS1, VWF	UCB exosomes in preeclampsia show significant proteomic alterations, with 29 differentially expressed proteins enriched in complement and coagulation pathways. These dysregulated protein signatures suggest a mechanistic link between exosomal cargo and the pathophysiology of preeclampsia.	Pathophysiological mediators of preeclampsia
Xueya Z. et al. (48)	Preeclampsia	Total= 38 mother/infant pairs, N = 18 neonates from PE pregnancies, N = 20 neonates from non-PE pregnancies	Preeclampsia group: 33.3w [± 1.07] Normotensive group: 39w [± 0.22]	At birth	Plasma (UCB)	Not determined	Exosomes: (Size: 30-200nm)	EV markers: CD9 (tetraspanin), TSG101 (ESCRT complex), ALIX (ESCRT complex), EV Cargo of interest: miR-125a-5p	Exosomal miR-125a-5p was significantly upregulated in both UCB & peripheral blood of women with preeclampsia, while its target VEGFA was downregulated in placental tissues. miR-125a-5p overexpression inhibited trophoblast migration, proliferation, and angiogenesis, suggesting a mechanistic contribution to preeclampsia.	Pathophysiological mediators of preeclampsia
Kunte P. et al.	Pregnancy compications	Total= 127 mother-infant pairs, N = 51 lean infants (lowest tertile of skinfold sum), N = 76 adipose infants (highest tertile of skinfold sum)	Lean neonates: mean GA: 32.5w, Adipose neonates: 32w	At birth	Plasma (UCB)	Adipocytes	Small Evs (Size: not determined)	EV markers: FABP4:(ositive selection of adipocyte-derived Evs), EV cargo: Differentially expressed miRNAs included: Maternal: miR-1202, miR-212-3p, miR-483-3p, miR-451a, etc. Cord blood: miR-204-5p, miR-483-5p, miR-3162-3p, miR-92a-3p, etc. Pathways regulated: FOXO, MAPK, TGF-β, Insulin, Wnt, JAK-STAT, Ras, etc.	Maternal and cord blood ADsEV miRNAs are correlated and differentially expressed in adipose versus lean neonates. Maternal ADsEV miRNAs targeted overlapping adipogenic pathways, suggesting a coordinated maternal–fetal signaling, modulated by maternal GDM, adiposity and micronutrient status, affecting neonatal adiposity.	Pathophysiology/ molecular mediators of maternal–fetal signaling influencing neonatal adipogenesis
Miranda J. et al. (37)	Pregnancy compications	Total= 30, N = 10 IUGR, N = 10 SGA, N = 10 control	Control: 40.1 [39.2-40.6], SGA: 39.4 [38.1-40], IUGR: 38.7 [37.6-40.1]	At birth	Plasma (UCB)	Placental	Exosomes (Size: Controls: 90 ± 17 nm, SGA: 85 ± 17 nm, IUGR: 81 ± 15 nm)	EV markers: CD63, TSG101, Flotillin-1, PLAP (placental, syncytiotrophoblast-specific marker, Negative marker: Grp94 (ER protein, confirms purity)	Placental exosome contribution (CD63^+^/PLAP^+^) to total circulating exosomes was significantly reduced in SGA and FGR compared with controls in both maternal and fetal plasma. The PLAP^+^ ratio correlated strongly with birthweight percentile, suggesting reduced release of placental exosomes in FGR.	Biomarkers of placental function and fetal growth status.
Menon R. et al. (19)	Pregnancy compications	Total= 45 neonates, N = 13 preterm neonates, N = 10 neonates born after PPROM followed by preterm delivery, N = 10 term neonates, N = 12 term neonates born with elective cesarian section from pregnancies not in labor (TNIL)	Term: 37-42w, Preterm: <37w,	At birth	Plasma (UCB)	Not determined	Exosomes (Size: 98–112 nm)	EV surface/identity markers: CD81, CD63, TSG101 (ESCRT complex), FLOT-1, ICAM-1 Annexin A5, Negative marker: GM130 (Golgi protein absent → confirms purity), EV cargo: Proteomic profiling (LC–MS/MS) revealed: 786 total proteins identified. Differentially expressed proteins varied by condition (e.g., pPROM, PTB, TL, TNIL).	Cord plasma exosomes show similar size, quantity, and core protein markers across term and preterm pregnancies, with only modest differences in proteomic cargo reflecting nonspecific inflammatory signals. Their characteristics and proteomic content do not reliably distinguish normal from adverse pregnancy conditions	Biomarkers of non-specific fetal inflammatory signaling
Bruschi M. et al. (34)	Pregnancy complications	Total= 6, N = 3 Term neonates, N = 3 Preterm neonates	Term > 37w, Preterm: 28–32 w	At birth	HUCMSCs culture supernatant	HUCMSCs	Microvesicles (Size: not derermined)	EV markers (identity markers): Mitochondrial associated proteins: ATP5B (ATP synthase β-subunit), COX5A (Cytochrome c oxidase subunit 5A), EV cargo: Proteomics (Orbitrap MS): 3253 proteins identified: 173 differentially expressed proteins between preterm vs term Preterm MVs enriched in: Oxidative phosphorylation proteins, TCA cycle enzymes (MDH2, aconitase, etc.), Electron transport chain subunits (Complex I–V), Functional cargo activity: Oxygen consumption. ATP synthesis (only in preterm MVs), Complex III activity differences	Microvesicles derived from preterm and term umbilical cord MSCs contained over 3,000 proteins but showed distinct proteomic clustering, with preterm MVs enriched in inflammatory and oxidative metabolism pathways. Preterm MVs uniquely demonstrated aerobic ATP synthesis, while term MVs consumed oxygen but did not produce ATP.	Pathophysiological/functional biomarkers reflecting the inflammatory and metabolic immaturity of preterm infants.
Simoncini S. et al. (29)	Pregnancy complications	Total= 47, N = 29 preterm neonates, N = 18 term neonates	Term: > 37 w Preterm: 24–35 w	At birth	UC-ECFCs	Endothelial cells	Endothelial MPs (Size: 0.1–1.0 μm (as defined by flow cytometry & TRPS)	EV markers: Annexin V^+^ (defining apoptotic/endothelial MPs)	Preterm-derived ECFCs display a senescence-associated secretory phenotype characterized by markedly increased release of endothelial microparticles, driven by SIRT1 deficiency and MKK6–p38MAPK–Hsp27 pathway activation. These EMPs propagate senescence to naïve endothelial cells and correlate inversely with gestational age.	Paracrine mediators that induce endothelial senescence.
Go H. et al. (52)	Respiratory system	Total= 73 preterm infants (GA <32 w), N = 39 CLD group, N = 34, NCLD group	Overall: <32 weeks, Non-CLD: 28w [± 0.3], CLD: 25w [± 0.3]	At birth & at 28 DOL	Serum (UCB & Peripheral blood)	Not recorded	Exosomes (Size: 120-130nm)	EV Markers: CD9, CD63 detected in pellets only (not in supernatant). EV Cargo (miRNAs): 62 identified miRNAs universally expressed in EVs. Key differential cargo: miR-21, miR-17, miR-20a, miR-25, miR-30c, miR-146a, etc. miR-21 specifically increased in CLD by DOL28.	Serum EV miR-21 is significantly elevated in infants who develop CLD by DOL28, while it decreases in non-CLD infants. EV miR-21 showed strong diagnostic performance for predicting CLD (AUC 0.850).	Potential diagnostic biomarker and pathophysiological mediator of CLD.
Lal C.V. et al. (15)	Respiratory system	Total= 80 neonates, N = 18 ELBW intubated at birth, N = 12 ELBW for the validation of miRNA findings of the previous group, N = 25 Preterm with established severe BPD at 36 weeks PMA, N = 25 Term or near-term neonates, intubated but without lung disease	Severe BPD: 25w [± 2], Controls: 37w [± 2]	At birth (within 6 hours of life) At 36 weeks PMA	Tracheal aspirates	Airway epithelial cells Neutrophils	Exosome (Size: 30-150nm)	EV markers: Epithelial exosomes: MUC4 antibodies. Neutrophil-derived exosomes: CD66 antibodies. EV cargo (miRNA & mRNA) EV miRNA cargo: miR-876-3p (decreased in severe BPD: top predictive biomarker). Additional miRNAs identified in discovery cohort (40 dysregulated; 32 validated). Target mRNAs: MCL1, RBBP6 (increased when miR-876-3p decreases)	Infants with severe BPD release increased numbers of smaller exosomes in tracheal aspirates, accompanied by a marked reduction in exosomal miR-876-3p detectable at birth. Loss of this miRNA is predictive and mechanistically causative for BPD, as gain-of-function *in vivo* restores alveolarization and reduces lung inflammation	Predictive biomarker & pathophysiology mediators of severe BPD.
Ransom M.A. et al. (17)	Respiratory system	Total= 34 premature neonates	Samples collected from 22w1d to 34w6d Late canalicular group: 22w1d – 26w6d, average 24.86 weeks Saccular group: 27w – 34w6d	Single timepoint (not determined)	Tracheal aspirates	Epithelial cells, Immune cells, Progenitor cells, Mesenchymal cells	Extracellular vesicles (Size: ~50–600nm)	EV markers: Tetraspanins: CD9, CD63, CD81 Epithelial markers: CD326 (EPCAM), CD133 (PROM1), CD142 (F3), ROR1, CD24, CD29, CD44, SSEA-4 Immune markers: CD3, CD14, CD40, CD41b, CD45, CD56, HLA-ABC, HLA-DRDPDQ	EVs were consistently detectable in tracheal aspirates from 22–35w GA, with infants in the late canalicular stage showing larger EV sizes and higher CD24^+^ EV levels. CD24^+^ and CD14^+^ EVs were significantly elevated in infants who went on to develop BPD, indicating developmental and disease-associated shifts in EV characteristics.	Biomarcers of lung maturation and potential mediators of early inflammatory pathways associated with BPD risk.
Kanei S. et al. (51)	ROP	Total= 47, N = 33 neonates with ROP, N = 14 control neonates without ROP	Treatment-requiring cases: 25w+5d [± 3d] Non-treatment-requiring cases: 29w+6d [± 3d] No ROP cases: 33w [± 2d]	At 34 weeks PMA	Tears	Not determined	Extracellular vesicles (Size: ≤200 nm)	EV markers: CD9 (strong signal), EpCAM (low, epithelial EV marker), CD147 (low), EV cargo (miRNAs): 380-miRNA Highly upregulated in ROP: miR-96, let-7a, miR-297, miR-520a-5p After birth-weight adjustment (27 significant miRNAs):Highest: miR-449a, miR-523, miR-342-3p. Biologically important network-associated miRNAs: miR-520a-5p (VEGF-centered angiogenesis network), miR-96, miR-340, miR-449	Tear-derived EVs in infants with treatment-requiring ROP display a distinct, strongly upregulated miRNA signature, with miR-520a-5p emerging as the key discriminating marker tightly linked to VEGF-centered angiogenesis networks. Machine-learning models showed high diagnostic accuracy, indicating that tear EV-miRNAs may serve as minimally invasive biomarkers for early identification of severe ROP.	Tear-EV miRNAs function as angiogenesis-linked biomarkers

CHD, Congenital Heart Disease; OD, Organ Dysfunction; NOD, Non-Organ Dysfunction; CPB, Cardiopulmonary Bypass; ICU, Intensive Care Unit; NET, Neutrophil Extracellular Trap; NEC, Necrotizing Enterocolitis; ECMO, Extracorporeal Membrane Oxygenation; PS, Phosphatidylserine; PECAM-1, Platelet Endothelial Cell Adhesion Molecule-1; VEGFR2, Vascular Endothelial Growth Factor Receptor-2; MadCAM-1, Mucosal Addressin Cell Adhesion Molecule-1; HDN, Hemolytic Disease of the Newborn; ABO-HDN, ABO Hemolytic Disease of the Newborn; Rh-HDN, Rhesus Hemolytic Disease of the Newborn; EMPs, Endothelial Microparticles; vWF, von Willebrand Factor; MPs, Microparticles; TF, Tissue Factor; GA, Gestational Age; UCB, Umbilical Cord Blood; LEVs, Large Extracellular Vesicles; PEVs, Platelet-Derived Extracellular Vesicles; TFPI, Tissue Factor Pathway Inhibitor; IQR, Interquartile Range; PDMP, Platelet-Derived Microparticles; ELISA, Enzyme-Linked Immunosorbent Assay; GPIIb/IIIa, Glycoprotein IIb/IIIa; IVH, Intraventricular Hemorrhage; CSF, Cerebrospinal Fluid; PHH, Post-Hemorrhagic Hydrocephalus; ABE, Acute Bilirubin Encephalopathy; NF-κB, Nuclear Factor Kappa-B; PI3K-AKT, Phosphoinositide-3-Kinase / Protein Kinase B Pathway; MAPK, Mitogen-Activated Protein Kinase; HIE, Hypoxic-Ischemic Encephalopathy; CNTN2, Contactin-2; BDNF, Brain-Derived Neurotrophic Factor; FiO_2_, Fraction of Inspired Oxygen; ASC, Apoptosis-Associated Speck-Like Protein Containing a CARD; IL-1β, Interleukin-1 Beta; PE, Preeclampsia; PLAP, Placental Alkaline Phosphatase; ER, Endoplasmic Reticulum; PPROM, Preterm Premature Rupture of Membranes; CS, Cesarean Section; HUCMSCs, Human Umbilical Cord Mesenchymal Stem Cells; MSCs, Mesenchymal Stem Cells; TCA, Tricarboxylic Acid Cycle; UC-ECFCs, Umbilical Cord Endothelial Colony-Forming Cells; TRPS, Tunable Resistive Pulse Sensing; SIRT1, Sirtuin-1; CLD, Chronic Lung Disease; DOL, Day(s) of Life; AUC, Area Under the Curve; ELBW, Extremely Low Birth Weight; BPD, Bronchopulmonary Dysplasia; EPCAM, Epithelial Cell Adhesion Molecule; PROM1, Prominin-1; F3, Coagulation Factor III (Tissue Factor); HLA, Human Leukocyte Antigen; ROP, Retinopathy of Prematurity; VEGF, Vascular Endothelial Growth Factor.

#### 3.4.1 EVs in the frame of pregnancy complications and fetal programming

Studies support that EVs are important components of maternal–fetal communication and participate in condition-specific pathophysiological processes across diverse pregnancy complications. In metabolic disorders, adipose tissue–derived EVs (ADsEVs) carry dysregulated miRNAs which are shared between maternal and fetal circulations and are strongly associated with maternal adiposity and gestational diabetes (43). Their miRNA cargo, targets key adipogenic and metabolic pathways such as insulin, Wnt, MAPK, FoxO, TGF-β, JAK–STAT and adipocytokine signaling (43). Dysregulated ADsEV miRNAs reflect maternal adipose tissue dysfunction, with a possible role for EV-mediated epigenetic signaling in fetal programming and in shaping neonatal adiposity and body composition (43). These alterations could affect early life and long-term metabolic risk.

Studies on placental insufficiency syndromes, report a comparable size and total concentration of UCB exosomes between small for gestational age (SGA), intrauterine growth restriction (IUGR) and control groups. However, the proportional contribution of placenta-derived exosomes (Placental alkaline phosphatase (PLAP^+^)/CD63^+^) to total exosomes in both maternal and fetal plasma was significantly reduced in pregnancies complicated by SGA and fetal FGR, with a graded decrease according to disease severity (37). Placenta-derived exosomes appear as functional mediators of placental–maternal–fetal crosstalk and highlight their potential utility as non-invasive biomarkers of placental function and fetal growth impairment (37).

Conversely, across term not in labor (TNIL), term in labor (TL), preterm birth (PTB), and preterm premature rupture of membranes (pPROM) cord blood exosomes show minimal differences in size, concentration, and protein cargo, indicating similar characteristics irrespective of pregnancy condition (19). Although proteomic analysis identified numerous cargo proteins, only a limited number were differentially abundant between groups. Pathway analyses primarily reflected nonspecific inflammatory and immune-related processes, with modest alterations in signaling pathways such as Wnt/β-catenin and corticotropin-releasing hormone signaling (19). In this study (19) UCB exosomes provide limited mechanistic insight into the pathophysiology of PTB or pPROM, although other EV subtypes or miRNA cargo may hold greater diagnostic potential. In contrast, microvesicles derived from fetal and neonatal cells appear to actively propagate postnatal vascular and inflammatory pathology associated with prematurity (34). Microvesicles released by HUCMSCs from preterm newborns exhibit enhanced mitochondrial and oxidative metabolism and have a distinct, metabolically active proteomic profile compared with term counterparts (34). From the proteomic cargo, 173 proteins were significantly altered and predominantly upregulated in preterm MVs, particularly those involved in oxidative phosphorylation and tricarboxylic acid cycle pathways. Functional assays show that, unlike term MVs, preterm MVs actively consume oxygen and generate ATP, indicating coupled oxidative metabolism, consistent with increased expression of respiratory chain and ATP synthase subunits (34). Similarly, endothelial progenitors from preterm neonates undergo stress-induced premature senescence with a pro-inflammatory secretory phenotype and markedly increased release of EMPs. EMP levels correlate with senescence and cytokine production, and functional assays demonstrate that EMPs transmit senescence and growth arrest to naïve endothelial cells via paracrine signaling (29). It is possible that senescent preterm endothelial cells actively propagate vascular dysfunction through EMP-mediated mechanisms, potentially contributing to persistent endothelial impairment after preterm birth (29). Collectively, EVs can function not only as biomarkers of placental and fetal status but also as active mediators of metabolic programming, placental insufficiency, and prematurity-associated vascular dysfunction.

Current literature regarding the role of EVs in preeclampsia (PE), report marked quantitative and qualitative alterations in circulating EVs, including increased levels of procoagulant MPs and higher concentrations of UCB exosomes. These alterations possibly reflect and the systemic pro-inflammatory, pro-thrombotic, and anti-angiogenic milieu of the disease (33). Preeclamptic pregnancies are associated with elevated circulating total MPs, activated platelet-derived MPs, leukocyte-derived MPs, and tissue factor–bearing MPs, together with enhanced phospholipid-dependent procoagulant activity (33). In UCB, MPs are even more abundant compared to maternal blood, with further increases in PE pregnancies, supporting enhanced fetoplacental vesicle release and placental contribution to systemic endothelial dysfunction.

Additionally, proteomic profiling of UCB exosomes in PE demonstrates altered protein and miRNA cargo enriched in complement, coagulation, inflammatory and angiogenic pathways (47, 48). Exosomal miRNA cargo is markedly altered, with enrichment of miRNAs targeting pathways critical for placental development and vascular homeostasis, including vascular endothelial growth factor (VEGF) and nuclear factor kappa-light-chain-enhancer of activated B cells (NF-κB) signaling (48). Differentially expressed exosomal proteins in UCB cluster around hemostatic regulators (e.g., von Willebrand factor (vWF), fibrinogen alpha chain (FGA), Protein S gene (PROS1)) (47), while dysregulated miRNAs-particularly upregulated miR-125a-5p-target VEGF signaling and inhibit trophoblast proliferation, migration, and endothelial angiogenesis. Together, these findings unravel a pathophysiologic role for EVs as active mediators in PE (48). EVs provide a link between placental dysfunction and maternal endothelial injury through the transfer of procoagulant, pro-inflammatory, and anti-angiogenic molecular signals responsible for the systemic manifestations of PE (33).

#### 3.4.2 EVs in neonatal hemostasis

Over the past years, studies have assessed the presence and procoagulant properties of EVs in neonates. Two studies by Hujacova et al. (25, 26), found no GA–related differences in UCB counts of PDEVs (CD41^+^CD36^+^), activated platelet EVs (CD41^+^CD62^+^), or endothelial EVs (CD31^+^CD105^+^) between preterm and term neonates. However, EVs from preterm infants exhibited reduced expression of platelet (CD36), platelet activation (CD62), and endothelial (CD31) surface markers. Given that receptor expression varies with GA (64), these phenotypic differences likely reflect the immaturity of the neonatal hemostatic system and impaired platelet cellular interactions, including platelet–leukocyte aggregation.

High circulating MPs are linked to increased prothrombotic risk as they enhance coagulation by exposing PS and sometimes TF, facilitating the binding of clotting factors, the formation of prothrombinase complex, and thrombin generation (65). Uszynski et al. (9) reported significantly higher procoagulant MPs in UCB compared with paired maternal plasma in term deliveries. Concurrently, they reported increased PS exposure and higher TF antigen alongside lower TF pathway inhibitor (TFPI) antigen. This indicates a predominance of procoagulant MP activity in fetal circulation. TF-bearing MPs may promote thrombin generation in the fetus by partially bypassing TFPI and thereby compensating for the physiologic immaturity and low fetal clotting factor levels (65). The absence of correlations between fetal and maternal MPs, TF, and TFPI levels suggests independent regulation within separate circulatory compartments, likely due to placental separation and developmental differences in MP production and clearance (9). While antigen-based findings support higher fetal TF availability, discrepancies with activity-based studies indicate that fetal TF may be functionally immature, warranting further functional and phenotypic studies. Korbal et al. (35) found significantly higher levels of TF-bearing MPs in UCB of preterm compared with term neonates and a negative correlation of TF-MPs with GA and birth weight, whereas total TF showed no such associations. Wasiluk et al. (36) showed that PDMPs were significantly higher in UCB of preterm versus term neonates, despite overall lower platelets in this group. This effect was particularly pronounced in male preterm infants and was independent of platelet counts. This observation is consistent with experimental data demonstrating sex-related differences in platelet responsiveness, whereby male platelets exhibit increased sensitivity to aggregating stimuli, likely mediated by androgenic hormones, and associated with an elevated thrombotic and cardiovascular risk (66).

Across studies, neonatal MPs exhibit qualitative features that can enhance hemostasis despite developmental immaturity of coagulation pathways. Functional assays in UCB samples demonstrated that, although MP counts may be similar to adults, neonatal MPs show greater procoagulant activity and stronger effects on thrombin generation, supporting a compensatory role in effective perinatal hemostasis without evidence of excessive platelet activation during normal delivery (23, 24, 67). Consistently, neonatal plasma exosomes are smaller but with a cargo profile that favors coagulation. They are enriched in procoagulant, platelet-related proteins (e.g., vWF, fibrinogen, FV, FVIII, PF4, and integrin signaling components) and relatively depleted of anticoagulant protein S and immunoglobulins (38). Neonatal platelets, particularly in the first days of life, also display higher baseline activation and increased MP formation, correlating with vWF binding and possibly driven by increased large vWF multimers (22).

However, MP quantity does not reliably predict procoagulant function, and results vary with GA, sampling site, and timing. Peripheral blood from healthy term neonates has shown lower MP procoagulant activity than adults, in contrast to UCB findings, suggesting that peripartum and placental contributions possibly influence cord MP activity (53). In preterm infants, platelet- derived microparticles (PDMPs) are more abundant but functionally less procoagulant due to impaired factor V/Va binding secondary to relative plasma factor V deficiency in this group (20). These data underscore that the immaturity of neonatal coagulation system affects the hemostatic impact of neonatal MPs with potential implications for bleeding risk and transfusion practices in preterm neonates. Overall, the studies suggest that neonatal hemostasis may be supported by compensatory procoagulant mechanisms, with preterm infants showing relatively increased thrombin generation and platelet activation despite an otherwise immature coagulation system (67–72,).

#### 3.4.3 EVs in HDN and non-hemolytic hyperbilirubinemia

Awad et al. (42) used flow cytometry to measure circulating CD144^+^ EMPs in neonates with ABO HDN, Rhesus HDN, and healthy controls, based on the premise that ABO incompatibility may directly damage the endothelium due to A and B antigen expression on endothelial cells. EMP levels and vWF antigen levels were higher in HDN than in controls and were significantly greater in ABO HDN than in Rhesus HDN. In addition EMPs increased progressively with the severity of hyperbilirubinemia, and decreased after treatment, most notably after exchange transfusion. Following this study, Zhu et al. (49) conducted a cohort of neonates with ABO HDN, Rh HDN, and healthy controls, on which pre-therapy CD144^+^ EMPs and vWF Ag levels were significantly elevated in both hemolytic groups compared with controls and were highest in ABO HDN. Similarly to the previous study, EMP levels decreased after treatment in both hemolytic groups and were independently associated with markers of hemolysis in ABO HDN, supporting their potential role as markers of disease severity and treatment response. Both studies propose potential mechanisms of endothelial injury in HDN but lack direct clinical assessment of endothelial dysfunction. In addition, evidence indicates that phototherapy can directly modulate endothelial cell function and activation by influencing angiogenic and redox-dependent signaling pathways (73, 74). Given the close relationship between these pathways and EV biogenesis, the potential impact of phototherapy on the reported EMP levels should be carefully considered. In terms of non-hemolytic hyperbilirubinemia, Yücesoy et al. (39) found that these neonates had significantly higher total and endothelial-derived apoptotic MP levels compared to controls, and these levels did not decrease after phototherapy despite the reduction in bilirubin concentration. This finding possibly aligns with cellular apoptosis induced by bilirubin-related oxidative stress, along with free radical generation during phototherapy (75, 76). In this context, EVs data suggest that HDN, hyperbilirubinemia, and phototherapy may influence neonatal vascular integrity and hemostatic balance, highlighting mechanisms that warrant further investigation. The differential response of EMPs after phototherapy between hemolytic and non-hemolytic neonatal jaundice may provide important insights not only into disease severity and treatment response, but also into the underlying pathophysiological mechanisms of neonatal jaundice. This observation could contribute to a better understanding of the pathogenesis of different types of hyperbilirubinemia and may have implications for both diagnostic evaluation and therapeutic management.

#### 3.4.4 EVs in critically ill neonates

Studies evaluate EVs as both indicators and potential mediators of pathophysiology in critically ill neonates. Term newborns receiving ECMO, exhibited significantly elevated systemic inflammatory markers, increased endothelial dysfunction markers (endocan and angiopoietin-2), reduced VEGF concentrations, and higher levels of circulating microvesicles compared with healthy controls, indicating widespread vascular and cellular injury (8). Total annexin-V–positive microvesicles and specifically mucosal endothelial (MadCAM-1–positive) microvesicles were significantly increased, suggesting preferential involvement of the intestinal microvasculature, likely related to hypoperfusion–reperfusion injury and possibly exacerbated by extracorporeal circulation itself (8). However, variation in the clinical conditions requiring ECMO may have influenced the results in this study. Moreover, in supported patients it remains challenging to separate EVs originating from extracorporeal circuit exposure from those driven by the underlying pathology (8, 77).

In premature infants with NEC and sepsis, urine-derived EVs carry disease-specific miRNA signatures linked to inflammatory and injury-related pathways involving key regulators such as TP53, TNF-α, and TGF-β, with concordant molecular alterations observed in experimental NEC models (14). In this context EVs function as mediators of systemic inflammation, endothelial injury, and organ-specific vulnerability in neonatal critical illness, while also serve as noninvasive biomarkers for disease characterization, progression, and potentially early risk stratification (8, 77).

In neonates and infants undergoing congenital cardiac surgery, plasma EV miRNA profiles clearly distinguished patients who developed postoperative organ dysfunction (OD) from those without OD across perioperative timepoints (21). Numerous differentially expressed miRNAs were already present at baseline and a distinct postoperative day-1 signature was enriched for pathways related to apoptosis, interferon signaling, infection, neutrophil extracellular trap (NET) formation, and complement/coagulation cascades. Proteomic analyses confirmed dynamic complement dysregulation in affected patients, characterized by baseline activation associated with tissue and endothelial injury followed by perioperative complement consumption (21). Integration of EV miRNA and protein data suggests that EV miRNAs may participate in post-transcriptional regulation of complement and inflammatory pathways, linking EV cargo to mechanisms of endothelial injury and multiorgan dysfunction and supporting their potential as mechanistic biomarkers in critically ill neonates (8, 14, 72).

**EVs in neonatal neurologic complications**.

Growing evidence indicates that EVs, are not only biomarkers of neonatal brain injury but also active mediators of neuropathological processes across diverse neonatal conditions (18). Across multiple neonatal disorders, EVs reflect and potentially propagate brain injury through condition-specific molecular cargo. In hypoxic ischemic encephalopathy (HIE), longitudinal changes in neuron-derived exosomal synaptopodin in peripheral blood correlate strongly with MRI injury severity, length of hospitalization, and need for anticonvulsant therapy. Thus it is supported that exosomes and their cargo could serve as dynamic indicators of neuronal injury and therapeutic response (18). In post-hemorrhagic hydrocephalus following Germinal Matrix Hemorrhage–Intraventricular Hemorrhage, CSF exosomes show evolving size distributions and shifting miRNA cargo over time. These changes are consistent with ongoing neurobiological and blood–brain barrier–related processes which reflect disease progression and recovery (40).

In acute bilirubin encephalopathy, CSF EVs display a proteomic signature enriched in immune, inflammatory, and oxidative stress pathways. This proteomic cargo, is suggestive of EV-mediated signaling in bilirubin-induced neurotoxicity and of candidate biomarkers and therapeutic targets (45). Exosomal neural proteins in UCB, including Contactin 2 (CNTN2) and brain-derived neurotrophic factor (BDNF), were assessed as biomarkers of brain health. Exosomal CNTN2 correlated positively with UCB ferritin and was especially sensitive to maternal diabetes, decreasing in males and increasing in females. BDNF showed inverse associations with ferritin and maternal anemia, more evident in females. It is proposed that exosomal CNTN2 and BDNF reflect neural responses to iron availability and metabolic stress *in utero*, linked to impaired neurodevelopment (16). Finally, in preterm infants exposed to prolonged oxygen therapy, alveolar macrophage–derived EVs enriched in apoptosis-associated speck-like protein containing a caspase recruitment domain (ASC), enter the circulation, cross the blood–brain barrier, and induce hippocampal inflammation and neural cell death based on experimental data. This EV-mediated lung–brain axis provides a mechanistic link between BPD and neurodevelopmental impairment (30). These studies indicate that EVs serve as integrative biomarkers of neural injury and systemic stress while also acting as biologically active vectors that can amplify inflammation, disrupt neurogenesis, and contribute directly to neonatal neurological adverse outcomes.

#### 3.4.5 EVs in neonatal chronic lung disease

Studies of circulating and airway EVs in preterm infants demonstrate disease-specific alterations in EV cargo rather than consistent changes in EV abundance. In serum samples, EV-miR-21 is significantly increased in preterm infants who develop CLD, particularly on day of life 28, where it serves as an independent risk factor with good diagnostic performance (AUC 0.85) (52). EV miR-21 was higher in severe CLD, in parallel with increased TGF-β, suggesting pathogenic miR-21–TGF-β signaling pathways in human CLD (52). miR-21 appears to drive maladaptive repair and fibrotic/inflammatory signaling in the developing lung, contributing to CLD/BPD pathogenesis, while also serving as a clinically relevant biomarker of disease risk and severity (78). Infants with severe BPD exhibit increased numbers of smaller epithelial-derived exosomes in the airways. In extremely low birth weight infants, reduced airway exosomal miR-876-3p at birth predicts subsequent severe BPD and is further decreased in established disease, correlating with disease severity (15). Functionally, miR-876-3p supports normal lung development and limits inflammation, and its restoration attenuates lung injury in experimental models. *In vitro* and murine models demonstrated that hyperoxia and lipopolysaccharide exposure decreased EV miR-876-3p levels, while restoration of miR-876-3p via intranasal mimic administration attenuated alveolar hypoplasia, supporting its potential role as a pathogenic mediator, biomarker, and therapeutic target in BPD. Thus, decreased exosomal miR-876-3p reflects early pathogenic mechanisms and represents a promising noninvasive biomarker for risk stratification in preterm infants (15).

Developmental stage also influences airway EV characteristics and biomarker profiles. EVs are detectable in tracheal aspirates from 22–35 weeks’ gestation and are enriched for epithelial markers (EPCAM/CD326, PROM1/CD133, and tetraspanins) (17). More specifically, larger EVs and higher CD24^+^ EV levels appear in the late canalicular stage, and elevated CD24^+^ and CD14^+^ EVs are present in infants who later develop BPD. Integration with single-cell atlases of developing lung epithelium supports developmental regulation of these EV markers (17). Functionally, miR-876-3p acts as a protective regulator of alveolar development and inflammation, with experimental restoration mitigating lung injury, whereas increased EV miR-21 is associated with profibrotic signaling (17, 79). Together, these findings indicate that EV surface markers and miRNA cargo reflect both developmental state and early pathogenic processes, supporting their utility as noninvasive biomarkers and potential therapeutic targets in neonatal chronic lung disease.

#### 3.4.6 EVs in retinopathy of prematurity

One study assessed the role of EVs and their miRNA cargo in tear fluid among preterm neonates as biomarkers of disease severity in ROP. EVs isolated from tear fluid contained distinct miRNA profiles that differentiated neonates with and without ROP and further discriminated treatment-requiring from non-treatment-requiring ROP, with 202 miRNAs initially identified as differentially expressed and a subset linked to angiogenic and inflammatory pathways (51). Machine-learning–based feature selection and validation identified miR-520a-5p as a promising biomarker for treatment-requiring ROP, demonstrating high diagnostic accuracy and a dose–response relationship with disease severity, although its expression was strongly confounded by GA, while other miRNAs (e.g., miR-378) showed gestational-age–independent associations. Network analyses implicated VEGF, NF-κB, ERK/MAPK, and IGF1R signaling, supporting a role for EV-miRNAs in retinal ischemia-driven angiogenesis and inflammation (51). Overall, the study supports tear-derived EV miRNAs as stable, minimally invasive biomarkers that may reflect local ocular pathophysiology in ROP, while emphasizing the need for larger, longitudinal studies to validate clinical utility and clarify independent mechanistic contributions.

### 3.5 Laboratory methods for EV assessment

The analytical methods used to assess EVs varied across the included studies, with differences observed in preanalytical sample handling, EV characterization procedures, evaluation of EV cargo, and the functional assays performed. (**Supplementary Table 4**) Circulating EVs were predominantly analyzed from plasma obtained from UCB or peripheral blood, with citrate being the most frequently used anticoagulant to preserve EV integrity and minimize *in vitro* coagulation and platelet activation (21, 33, 36, 53). Additional biological matrices included urine (14), placental tissue (34, 44) and cell culture supernatants (41) typically collected in sterile tubes. Initial processing generally involved sequential low- and intermediate-speed centrifugation to remove cells and debris and to generate platelet-poor or platelet-free plasma, with some studies incorporating filtration or repeated centrifugation steps to further reduce contaminating platelets and protein aggregates (18, 42, 51).

Several studies explicitly reported temperature-controlled processing to preserve EV integrity and limit post-collection vesiculation, including centrifugation steps performed at 4 °C (17, 31, 45) and storage of processed samples at −80 °C prior to downstream analyses (17, 51). EV/MP isolation strategies varied substantially and included differential ultracentrifugation (29, 34), size-exclusion chromatography (SEC) (40, 43), polymer-based precipitation methods (45, 47) and membrane affinity or column-based commercial kits optimized for downstream RNA or proteomic analyses (21, 31). In selected studies, immunoaffinity approaches were applied to enrich EVs expressing specific surface antigens (18, 35).

Phenotypic characterization was most commonly performed using flow cytometry with fluorescently labeled antibodies (27, 28, 33, 42, 53). Annexin V binding was frequently used to detect PS exposure and to identify procoagulant EVs (9, 33, 53). PDEVs were primarily identified using CD41, CD42, CD61, and CD62P (30, 33, 42, 53). Endothelial-derived EVs were characterized using markers such as CD144, CD31, CD105, and CD34 (11). Leukocyte-derived EVs were less frequently assessed and were mainly identified using CD45 (11).

Established EV markers were commonly evaluated in studies focusing on SEVs or exosomes. These included CD63, CD9, CD81, TSG101, ALIX, and HSP70 (14, 29, 31, 34). These markers were detected using flow cytometry, Western blotting, or bead-based immunoassays, depending on the analytical platform (31, 34). TF expression (CD142) was assessed in selected studies investigating thrombotic mechanisms and EV-associated coagulation activity (33, 53). In these studies, TF^+^ EVs were frequently analyzed together with PS^+^ populations to estimate procoagulant potential (9, 33). High-sensitivity or small-particle flow cytometry platforms were used in some studies to improve detection of submicron EVs (8, 30).

EV size distribution and concentration were assessed using nanoparticle tracking analysis (NTA) (46, 47, 49), dynamic light scattering, or flow cytometry-based particle counting, depending on the targeted EV size range. NTA was frequently employed to quantify total EV concentration and to generate size distribution profiles (45, 47, 49), whereas flow cytometry was used to provide subtype-specific counts based on antigen expression (27, 28, 33, 42, 53). In some studies, protein-based quantification assays were used as surrogate measures of EV abundance (e.g., total protein by BCA or Bradford assays) (29, 34), although these approaches do not discriminate vesicular from non-vesicular protein components.

Cargo analysis encompassed nucleic acids, proteins, and metabolites. RNA profiling, EV-associated miRNAs and other small RNAs were extracted using membrane-affinity or phenol-based methods and were analyzed by quantitative reverse transcription polymerase chain reaction (qRT-PCR), microarray platforms, or next-generation sequencing (NGS) (14, 21, 31, 48). Proteomic analyses was performed using mass spectrometry-based approaches, most commonly liquid chromatography-tandem mass spectrometry (LC–MS/MS), following EV lysis and enzymatic protein digestion (29, 34, 40). Targeted protein quantification was additionally performed using enzyme-linked immunosorbent assays (ELISA) or Western blotting to measure selected inflammatory, endothelial, or coagulation-related molecules (23, 33, 53). Metabolomic analyses were less frequently reported and were conducted using mass spectrometry to characterize metabolic signatures associated with EV populations (40, 46).

Functional assessment of EVs procoagulant activity was conducted in several studies using phospholipid-dependent clotting assays, thrombin generation assays, or commercial procoagulant phospholipid (PPL) activity tests (9, 33, 53). Cellular functional assays were used to evaluate endothelial activation, mitochondrial function, or metabolic effects were investigated using cellular bioenergetic measurements, oxygen consumption assays, or *in vitro* cell activation models following EV exposure (29, 34, 40). Morphological validation of EVs was less consistently reported but, when performed, relied primarily on transmission electron microscopy to confirm vesicular structure and size (8, 14, 34).

### 3.6 Methodological limitations related to EV quantification and detection

Interpretation and comparison of EV-related outcomes across studies is limited by substantial heterogeneity in EVs isolation, detection, and quantification methodologies. Studies employed diverse techniques, including NTA, conventional or high-sensitivity flow cytometry, and protein-based surrogate assays, each of which measures different physical or biochemical properties and targets partially overlapping EV populations. In addition, several approaches, particularly protein-based quantification methods, do not discriminate vesicular from non-vesicular components, potentially leading to overestimation of EV abundance (3, 29, 34) Notably some of the included studies do not report a precise separation of EVs from NVEPs (8, 9, 15, 16, 18, 20–24, 26, 29, 33–36, 39, 42, 43, 45, 47, 49–53). However, even studies that adhere more closely to the MISEV guidelines are unable to achieve complete discrimination between EVs and NVEPs, a limitation that is also acknowledged in some of these works. In the final column of **Supplementary Table 4**, we therefore have presented those studies that followed, to the greatest extent possible, the recommended laboratory approaches for improving the separation and characterization of EVs relative to NVEPs, as outlined by MISEV (11, 14, 17, 19, 25, 27, 28, 30–32, 37, 38, 40, 41, 44, 46, 48).

Pre-analytical variability, in anticoagulant choice, centrifugation protocols, filtration steps, storage temperature, and freeze–thaw cycles, further contributes to inter-study variability and may influence both EV yield and phenotype. Moreover, limited use of orthogonal characterization techniques and inconsistent reporting of methodological details restrict reproducibility and hinder assessment of EV purity and subtype specificity (3).

Pre-analytical handling markedly influences platelet EV measurements. Delayed processing of citrated whole cord blood induces artificial increases in EV counts due to *in-vitro* platelet activation, whereas early plasma separation with short, refrigerated storage seems to have preserved EV counts and fluorescence (25, 26). In addition, hemolysis represents an important confounder, as samples with elevated free hemoglobin showed more than a three-fold increase in platelet and activated platelet EV counts, indicating a predominantly quantitative bias (25, 26). Hemolysis may reflect platelet activation or unrecognized handling artifacts. There is need for strict control of processing time, avoidance of freezing, and routine monitoring of free hemoglobin to ensure reliable EV assessment.

These limitations are consistent with concerns highlighted in the MISEV guidelines, which recommend the use of multiple complementary methods for EV characterization, transparent reporting of pre-analytical variables, and avoidance of single-parameter quantification strategies (3). The lack of uniform adherence to these recommendations in the included studies underscores the need for standardized workflows to improve comparability, reproducibility, and biological interpretation of EV-related findings. Given the variability in analytical methodologies and outcome reporting, quantitative synthesis was not feasible, and findings were therefore presented using a qualitative synthesis approach.

### 3.7 Risk of bias assessment

Risk of bias assessment of the included studies was performed using the Newcastle Ottawa scale for Cross sectional, case-control, and prospective cohort studies (80–82). The methodological quality of the included studies generally reflects a moderate-to-high standard across the various study designs, with particular strengths observed in case definition and exposure ascertainment. Case-control studies exhibited high consistency, with scores ranging from 7 to 8 stars, indicating robust selection of controls and precise case definitions in works such as those by Vítková V. et al. and Awad H.A. et al (8, 42). In contrast cross-sectional and prospective cohort studies showed broader variability, both ranging from 5 to 9 stars. While some studies achieved the maximum score of 9 stars such as the study of Lal C.V. et al. (15), common limitations identified across the literature. These included insufficient justification of sample sizes in cross-sectional designs, as seen in Huang S. et al., and challenges in ensuring adequate long-term follow-up in longitudinal cohorts like that of the study of Goetzl L. et al (18). (**Supplementary Tables 1**-**3**).

A significant discrepancy exists between the high-quality scores assigned via the NOS scale and the methodological limitations revealed when applying the MISEV guidelines (3). While the NOS focuses on epidemiological rigor, it does not account for the specialized technical challenges of EV research, such as the frequent lack of precise separation between EVs and NVEPs noted in many works, including studies by Xueya Z. et al. (48), and Spaull R. et al (40). Many studies that scored well on the NOS utilized single-parameter quantification strategies or protein-based surrogate assays (e.g., BCA or Bradford) that do not discriminate vesicular components, which can lead to an overestimation of EV abundance. This limitation is explicitly acknowledged or present in the methodologies of Bruschi M. et al. (34), Simoncini S. et al (29)., and Xueya Z. et al. (48), where total protein levels were often used as a proxy for EV concentration. This underscores a paradox where a study may be epidemiologically “high quality” according to the NOS but technically limited by MISEV standards due to pre-analytical variability in anticoagulants, centrifugation protocols, and a lack of orthogonal characterization techniques.

## 5 Discussion and future directions

**Figure 4:**
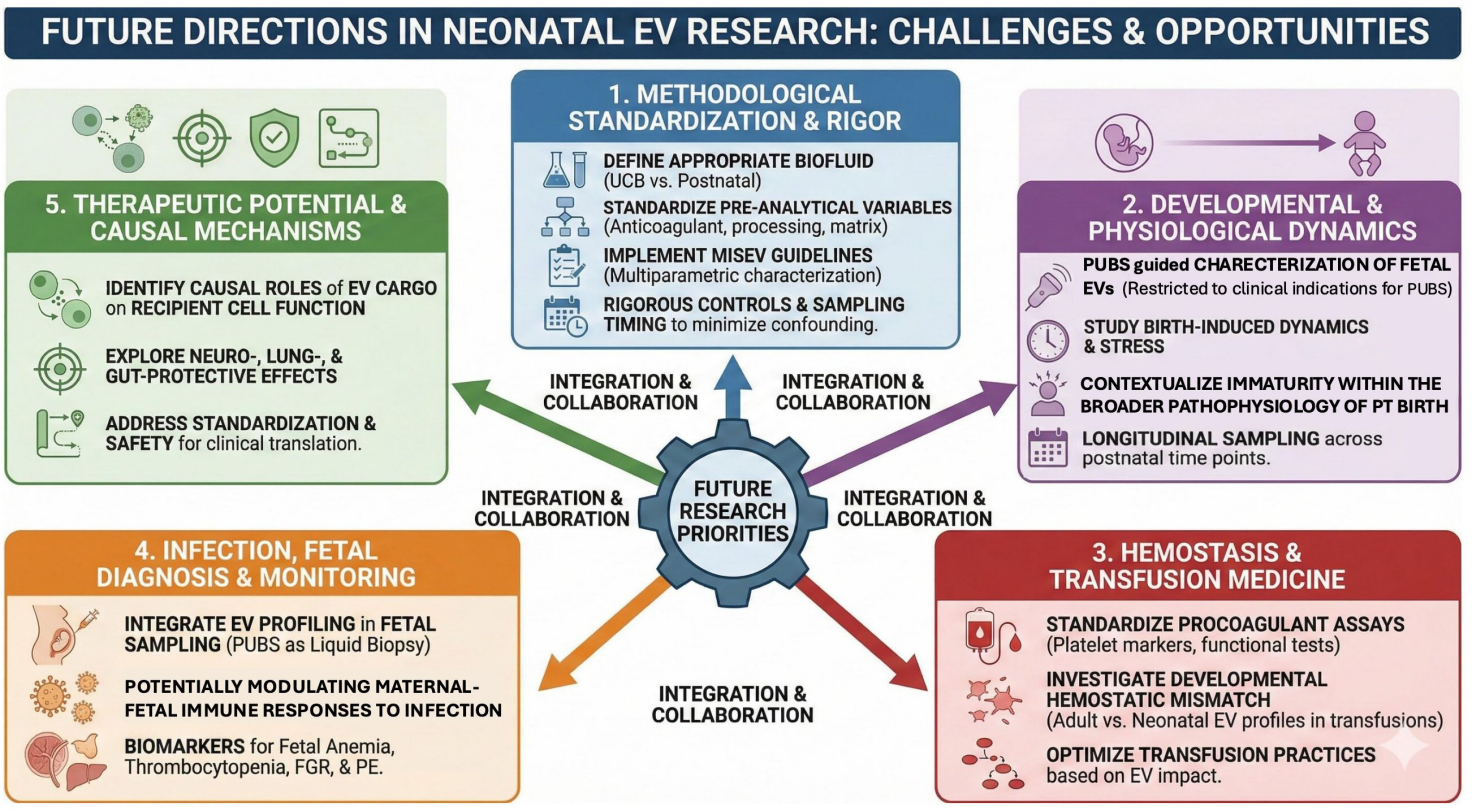
Future directions in EV research in neonates. It highlights five interconnected priorities: achieving methodological standardization, understanding developmental and physiological dynamics, investigating roles in hemostasis and transfusion medicine, exploring applications in infection, fetal diagnosis, and monitoring, and elucidating therapeutic potential and causal mechanisms. The central gear emphasizes the necessity of integration and collaboration across these distinct but related areas to advance the field. EV, Extracellular Vesicle; UCB, Umbilical Cord Blood; MISEV, Minimal Information for Studies of Extracellular Vesicles (guidelines); PUBS, Percutaneous Umbilical Blood Sampling; FGR, Fetal Growth Restriction; PE, Preeclampsia. (Image created with Gemini based on the text provided in Section 5. Discussion and Future directions).

To our knowledge, the present systematic review is the first to compile and qualitatively synthesize the existing evidence regarding the biological role and clinical implications of EVs in neonates. Despite the small to moderate sample sizes and the heterogeneity of clinical and laboratory settings, the available data demonstrate the consistent presence and biological activity of EVs across a range of neonatal conditions. Overall, these findings underscore the need for larger, standardized, and multiparametric studies to elucidate the biological functions of EVs and to further support their potential role in the diagnosis, prognosis, and therapeutic management of neonatal diseases (**Figure 4**).

The findings of this review underscore that neonatal EVs function as integral components of a complex, adaptive intercellular communication network that orchestrates extrauterine transition (11, 32). Across the included studies, EVs derived from multiple cellular sources—including platelets, endothelial cells, leukocytes, stem cells, epithelial cells, placental tissues, and microbial communities—carried highly diverse molecular cargo composed of miRNAs, proteins, lipids, coagulation mediators, inflammatory molecules, and metabolic signals (8, 27, 28, 32, 36, 37, 42, 43). Rather than acting as isolated biomarkers, these EV populations appear to function collectively as dynamic signaling hubs that integrate immune, vascular, metabolic, and developmental pathways during neonatal adaptation. Their cargo profiles consistently reflected coordinated regulation of innate immunity, endothelial activation, hemostasis, angiogenesis, oxidative stress responses, and tissue remodeling, indicating that EV-mediated communication contributes to the establishment of neonatal immune homeostasis while simultaneously responding to inflammatory or pathological stimuli. This systems-level organization becomes particularly evident in prematurity and neonatal disease, where altered EV composition and signaling signatures suggest disruption of tightly regulated intercellular networks linking the placenta, immune system, vasculature, lungs, gut, and brain (11, 18, 30, 37, 42, 49). The observed interactions between EV-associated coagulation pathways, inflammatory cascades, microbiome-derived signals, and organ-specific injury responses further support the concept that neonatal adaptation is governed by interconnected biological circuits rather than isolated organ processes. Within this framework, EVs emerge not only as biomarkers of disease states but also as active systems-level regulators capable of shaping immune maturation, inflammatory balance, vascular integrity, and developmental programming during the critical neonatal period.

Importantly, although many studies included in this review describe their findings in terms of “EV cargo” and EV-mediated biological effects, a substantial proportion of the available neonatal literature relied on isolation and characterization approaches that are unable to fully discriminate EVs from NVEPs, lipoproteins, protein aggregates, and other contaminants. Consequently, several reported molecular signatures and functional properties attributed to EVs may in fact reflect mixed extracellular particle populations rather than purified vesicular fractions. Therefore, the biological interpretation of EV cargo composition, intercellular signaling functions, and disease-specific mechanistic pathways derived from these studies must be approached with considerable caution. This limitation is not unique to neonatal research but remains a major challenge across the EV field, particularly in studies predating the updated MISEV2023 recommendations. Rigorous adherence to standardized multiparametric characterization strategies and improved separation methodologies will be essential before definitive conclusions regarding neonatal EV-specific biology and function can be reliably established.

Future studies should prioritize defining the most appropriate biofluid for neonatal EV analysis and clarifying the comparability of UCB samples and early postnatal samples. Greater standardization of pre-analytical variables, including anticoagulant selection, processing timelines, sample matrix, and isolation protocols, together with consistent implementation of MISEV-recommended multiparametric characterization, will be essential to improve reproducibility and data comparability. In addition, rigorous selection and reporting of control populations and precise documentation of sampling time points are needed to minimize confounding effects, thereby strengthening the interpretability and translational relevance of neonatal EV research (3).

Developmental changes in EV formation across gestation remain poorly characterized, largely due to limitations in fetal blood sampling. Ultrasound-guided umbilical cord puncture, currently used for prenatal diagnostics in mid gestation, could also enable characterization of EV abundance and cargo during fetal development. Although fetal EV biogenesis and regulation remain largely unexplored, birth appears to induce dynamic changes in EV quantity and phenotype, potentially driven by a labor associated stress. The molecular mechanisms underlying these adaptations are not fully understood. In preterm delivery, it remains unclear whether EV profiles reflect the pathophysiology of preterm birth or developmental immaturity alone.

Standardized, multiparametric approaches are essential to characterize the procoagulant properties of neonatal EVs, incorporating multiple platelet markers and complementary functional assays in accordance with MISEV recommendations. EVs should also be examined in the context of developmental hemostasis through longitudinal sampling across defined postnatal time points and correlation with clinically relevant outcomes, such as hemorrhage or thrombosis. Analysis of intrauterine UCB samples would elucidate the role of EVs in hemostatic maturation. Integrating rigorous EV identification with functional evaluation and clinical correlation will be essential to clarify the role of EVs in neonatal hemostasis and to resolve inconsistencies across existing studies.

Neonatal plasma exosomes are enriched in procoagulant proteins relative to adult exosomes, reflecting developmental adaptation of the neonatal hemostatic system (38). Transfusion of adult-derived platelet products, which contain exosomes with lower procoagulant potential, may perturb this physiological balance and contribute to adverse outcomes in preterm infants, consistent with a developmental hemostatic mismatch hypothesis (83, 84). This concept is supported by experimental evidence demonstrating that adult platelet transfusion alters neonatal hemostatic function *in vitro (*85). A similar mismatch may also be relevant to RBC transfusions in neonates, as adult donor RBC products contain bioactive EVs, membrane fragments, and storage-related inflammatory mediators that may influence endothelial activation, coagulation, and immune responses in the immature neonatal circulation (86). Assessment of EVs could deepen our understanding of the impact of adult blood products on the neonatal circulation and inform optimization of neonatal transfusion practices including blood component processing and storage conditions. Future studies should also compare EV profiles in UCB–derived versus adult donor transfusion products to elucidate age-specific differences and their potential clinical implications.

EVs are increasingly implicated in infection-related pregnancy complications associated with pathogens such as Zika virus, CMV, SARS-CoV-2 and listeria monocytogenes (87). Experimental evidence indicates that placental EVs released during bacterial and viral infections exhibit altered RNA and protein cargo capable of modulating inflammatory responses and transporting viral components to fetal cells (88–90). potentially facilitating vertical transmission and contributing to adverse pregnancy and neurodevelopmental outcomes. Integrating EV profiling into fetal and placental sampling strategies could enable early detection of vertical transmission risk, improve mechanistic understanding of infection-driven placental dysfunction, and support the development of EV-based biomarkers or targeted therapeutic interventions to mitigate adverse pregnancy and neurodevelopmental outcomes.

Building on the demonstrated clinical value of percutaneous UCB sampling (PUBS) for direct access to fetal circulation (91), the analysis of EVs in PUBS-derived samples represents a promising translational extension. EVs carry cell-specific proteins, lipids, and nucleic acids that reflect the functional and pathological state of their tissue of origin (92). In fetal anemia and during intravascular transfusion, EV profiling could provide real-time biomarkers of erythropoietic stress, hypoxia, inflammation, and transfusion-related cellular activation, potentially improving risk stratification and therapeutic monitoring. In fetal thrombocytopenia, EVs in fetal blood could act as biomarkers of platelet activation and immune-mediated platelet destruction, complementing standard platelet counts (93). Endothelial-derived EVs may additionally indicate endothelial injury and vascular instability, potentially identifying increased hemorrhagic risk. In FGR and PE, where placental insufficiency, hypoxia, and metabolic dysregulation dominate (37), EV cargo may capture placental–fetal signaling alterations and serve as early indicators of disease severity and fetal compromise. For fetal thyroid disorders, EVs derived from thyrocytes or immune cells may reflect thyroid functional status and immune-mediated mechanisms (94), offering a complementary molecular readout beyond hormone measurements, while in fetal cardiac conditions and arrhythmias, EVs may mirror myocardial stress, vascular remodeling, and response to intrauterine therapy. Collectively, despite PUBS being an invasive method that carries fetal loss risk and is restricted to clinical indications, integrating EV analysis with PUBS could transform this invasive but highly informative procedure into a molecular liquid biopsy platform. This could enable mechanistic insights, refined diagnosis, and longitudinal monitoring of fetal disease and therapeutic efficacy, provided that methodological standardization and validation studies are established.

The identification of causal roles of EV abundance and cargo in various neonatal clinical conditions is a key priority. Future research should focus on uncovering the biological impact of the molecular EV cargo on the recipient cell function in a context and tissue specific manner. Furthermore, these findings should be assessed within a longitudinal context in association with future clinical outcomes. Early experimental and emerging clinical data indicate meaningful neuroprotective (95), lung-protective (15), and gut-protective effects (96). However, clinical translation is currently limited by incomplete molecular characterization of EV cargo, variability in isolation and dosing methods, and unresolved safety concerns. Ongoing early-phase clinical trials and efforts toward standardized manufacturing and safety assessment suggest that EV-based therapies could become transformative for neonatal care once these barriers are addressed.

## 6 Limitations

The present systematic review is subject to certain limitations, mainly due to the substantial heterogeneity of the available studies which rendered a meta-analysis methodologically inappropriate and necessitated a qualitative synthesis of the data. Additionally, the overall number of studies focusing exclusively on the neonatal population remains limited. Consequently, the reliance on a qualitative synthesis of the available data necessitates cautious interpretation of the findings. Regarding certainty-of-evidence assessment, a GRADE certainty-of-evidence assessment tool was not conducted, as the majority of included studies did not adhere to MISEV guidelines, thereby limiting the feasibility and interpretability of such an analysis.

The fact that the search strategy was structured around comprehensive EV-related terminology rather than individual EV subtypes such as “exosome(s),” and was based on two databases could be considered as a limitation. However, the combination of EV-related terminology, two major databases such as PubMed and Scopus, and extensive snowballing successfully identified multiple exosome-focused studies, supporting the adequacy and robustness of the overall retrieval strategy. Despite these limitations, a major strength of the present study is that, according to our current knowledge, it represents one of the first systematic reviews to focus exclusively on the role of EVs in neonates. Given that neonates constitute a biologically distinct population, findings from adult studies cannot be directly interpreted to neonatology. The necessity to qualitatively synthesize and present our data, has allowed the article to dive in to deep pathophysiological pathways. From this perspective, the present work makes a substantial contribution by consolidating and critically appraising the existing evidence and by highlighting important gaps that warrant further investigation.

Although the majority of the studies included use the term EVs, the methodologies applied often do not allow for the precise discrimination of vesicular particles from NVEPs. The MISEV2023 guidelines provide a detailed information on the strengths and limitations of the currently available isolation and characterization approaches with regard to their ability to separate EVs from NVEPs and propose methodological strategies that can improve the rigor of EV enrichment and characterization. They also emphasize that complete separation of EVs from NVEPs is rarely achievable in complex biological samples (3). Given the very limited number of studies on EVs in neonates, we deliberately chose to include studies in which EVs were not clearly distinguished from NVEPs. In strict accordance with the MISEV recommendations, the more accurate term for such preparations would be “*extracellular particles”* or ‘*EV-containing preparations*”, however, we retained the terminology used by the original authors to avoid misrepresentation of the primary literature and to ensure consistency with the nomenclature employed in the respective publications, the majority of which predate the updated 2023 guidelines. While this constitutes a limitation of the present work, it also highlights the urgent need for methodological standardization and closer adherence to the ISEV/MISEV recommendations in future studies, in order to enable more accurate isolation, characterization, and reporting of EVs, as well as the consistent use of appropriate terminology.

## 7 Conclusions

Accumulating evidence underscores the multifaceted role of EVs in neonatal physiology and disease. The important contribution of EVs to haemostatic balance can be reflected through detected alterations in the concentrations of PDEVs, TF–bearing EVs, and enhanced procoagulant activity in the included studies. In parallel, emerging data support the utility of EV-associated molecular signatures as clinically relevant biomarkers, including specific miRNAs and protein markers linked to neonatal morbidities and short-term outcomes. These findings highlight the potential of EV profiling to improve risk stratification and disease monitoring during critical periods such as prematurity and perinatal adaptation. Beyond their diagnostic value, EVs and especially MSC–derived EVs, are increasingly recognized as promising therapeutic candidates for a spectrum of neonatal disorders. However, substantial gaps remain in the comprehensive molecular characterization of EV cargo and in the elucidation of their mechanisms of action. Future progress will depend on adequately powered studies which will employ standardized and rigorous EV isolation and characterization methodologies in line with MISEV recommendations. Correlating EV phenotypes with clinically meaningful endpoints will be essential to ensure reproducibility and translational validity. In parallel, well-controlled preclinical investigations addressing safety, dosing, and biological activity are required to support the responsible advancement of EV-based diagnostics and therapies into neonatal clinical practice.

## Data Availability

The original contributions presented in the study are included in the article/**Supplementary Material**. Further inquiries can be directed to the corresponding author.
