# Supplementary material for: Tiny messengers, big impact: unlocking the power of extracellular vesicles in neonatal health and disease. a systematic review
Source: Front Immunol. 2026 Jun 22;17:1848637. doi: 10.3389/fimmu.2026.1848637 (PMC13333448; doi:10.3389/fimmu.2026.1848637)
Supplement: Supplementary file 1 [file Table1.docx]

**Search strategy**

**1. Keywords**

Extracellular Vesicles, Extracellular vesicle, Microvesicles, Microparticles, Microparticle, Microvesicle, Neonate, Neonatal, Neonates, Newborn, Perinatal

**2. Search Algorithm (7/8/2025)**

**PubMed:** ("extracellular vesicles"[Title/Abstract] OR "extracellular vesicle"[Title/Abstract] OR "microvesicles"[Title/Abstract] OR "microvesicle"[Title/Abstract] OR "microparticles"[Title/Abstract] OR "microparticle"[Title/Abstract]) AND ("neonate"[Title/Abstract] OR "neonates"[Title/Abstract] OR "neonat*"[Title/Abstract] OR "newborn"[Title/Abstract] OR "newborns"[Title/Abstract] OR "perinatal"[Title/Abstract])

**Scopus:** TITLE-ABS-KEY ((“extracellular vesicles" OR "extracellular vesicle" OR "microvesicles" OR "microvesicle" OR "microparticles" OR "microparticle”) AND (“neonate" OR "neonates" OR "neonat*" OR "newborn" OR "newborns" OR "perinatal”))

**3. Databases and Results**

1. **PubMed:** 497 articles
2. **Scopus:** 935 articles

- **Total:** 1,432 articles
- 33 articles selected for inclusion

**4.** **Updated Search (08/08/2025 to 10/11/2025):**

1. **PubMed:** 28 articles
2. **Scopus**: 39 articles

- **Total:** 67 articles
- 1 article selected for inclusion

**5.** **Additional Sources:**

- **Snowball effect** (Detailed search of the references of the included studies, and of the excluded systematic and narrative reviews relevant to this study) : 9 articles included

**6. Final Total:** 43 articles included in the study
