# Supplementary material for: Tiny messengers, big impact: unlocking the power of extracellular vesicles in neonatal health and disease. a systematic review
Source: Front Immunol. 2026 Jun 22;17:1848637. doi: 10.3389/fimmu.2026.1848637 (PMC13333448; doi:10.3389/fimmu.2026.1848637)
Supplement: Supplementary file 2 [file Table2.docx]

|  | **Selection** | | | | **Comparability** | | **Exposure** | | | **Total** |
| --- | --- | --- | --- | --- | --- | --- | --- | --- | --- | --- |
| **Study** | **Case definition** | **Representativeness of the cases** | **Selection of controls** | **Definition of controls** | **On (The most important factor)** | **On additional factors** | **Ascertainment of exposure** | **Same method of ascertainment in cases and controls** | **Non-response rate** | 10x |
| Vítková V. et al. |  |  |  |  |  |  |  |  |  | c  7x |
| Jia R. et al. |  |  |  |  |  |  |  |  |  | 7x |
| Campello E. et al. |  |  |  |  |  |  |  |  |  | 7x |
| Awad H.A. et al. |  |  |  |  |  |  |  |  |  | 7x |

**Supplementary table 1. Newcastle-Ottawa scale for Case-Control Studies**

**Supplementary table 2. Newcastle-Ottawa scale for Cross-sectional studies**

|  | **Selection** | | | | **Comparability** | **Outcome** | | **Total** |
| --- | --- | --- | --- | --- | --- | --- | --- | --- |
| **Study** | **Representativeness of the sample** | **Sample size** | **Non-respondents** | **Ascertainment of the exposure (risk factor)** | **Comparability of subjects in different outcome groups on the basis of design or analysis. Confounding factors controlled.** | **Assesment of outcome** | **Statistical test** | **10x** |
| Galley J.D. et al. |  |  |  |  |  |  |  | \| 6x \| \| --- \| |
| Huang S. et al. |  |  |  |  |  |  |  | \| 5x \| \| --- \| |
| Hujacova A. et al. (2020) |  |  |  |  |  |  |  | \| 6x \| \| --- \| |
| Hujacova A. et al. (2021) |  |  |  |  |  |  |  | \| 6x \| \| --- \| |
| Kanei S. et al. |  |  |  |  |  |  |  | \| 8x \| \| --- \| |
| Karlaftis V. et al. |  |  |  |  |  |  |  | \| 6x \| \| --- \| |
| Korbal P. et al. |  |  |  |  |  |  |  | \| 8x \| \| --- \| |
| Kunte P. et al. |  |  |  |  |  |  |  | \| 9x \| \| --- \| |
| Marell P. et al. |  |  |  |  |  |  |  | \| 8x \| \| --- \| |
| Michelson A.D. et al. |  |  |  |  |  |  |  | \| 6x \| \| --- \| |
| O'Reilly D. et al. |  |  |  |  |  |  |  | \| 6x \| \| --- \| |
| Peñas-Martínez J. et al. |  |  |  |  |  |  |  | \| 8x \| \| --- \| |
| Ramkumar M. et al. |  |  |  |  |  |  |  | \| 7x \| \| --- \| |
| Schmugge M. et al. |  |  |  |  |  |  |  | \| 7x \| \| --- \| |
| Schweintzger S. et al. (2010) |  |  |  |  |  |  |  | \| 7x \| \| --- \| |
| Schweintzger S. et al. (2011) |  |  |  |  |  |  |  | \| 7x \| \| --- \| |
| Starke N. et al. |  |  |  |  |  |  |  | \| 8x \| \| --- \| |
| Tan N. et al. |  |  | \|  \| \| --- \| |  |  |  |  | \| 7x \| \| --- \| |
| Turunen J. et al. (2021) |  |  |  |  |  |  |  | \| 6x \| \| --- \| |
| Turunen J. et al. (2023) |  |  |  |  |  |  |  | \| 6x \| \| --- \| |
| Uszynski M. et al. |  |  |  |  |  |  |  | \| 7x \| \| --- \| |
| Wang D. J. et al. |  |  |  |  |  |  |  | \| 7x \| \| --- \| |
| Wasiluk A. et al. |  |  |  |  |  |  |  | \| 7x \| \| --- \| |
| Xagorari A. et al. |  |  |  |  |  |  |  | \| 7x \| \| --- \| |
| Yücesoy E. et al. |  |  |  |  |  |  |  | \| 7x \| \| --- \| |
| Zhu X.J. et al. |  |  |  |  |  |  |  | \| 8x \| \| --- \| |
| Xueya Z. et al. |  |  |  |  |  |  |  | \| 7x \| \| --- \| |
| Keller S. et al. |  |  |  |  |  |  |  | 7x |
| Bruschi M. et al. |  |  |  |  |  |  |  | 7x |
| Simoncini S. et al. |  |  |  |  |  |  |  | \| 8x \| \| --- \| |

**Supplementary table 3. Newcastle-Ottawa scale for Prospective cohort studies**

|  | **Selection** | | | | **Comparability** | | **Outcome** | | | **Total** |
| --- | --- | --- | --- | --- | --- | --- | --- | --- | --- | --- |
| **Study** | **Representativeness** | **Selection of non-exposed** | **Ascertainment of exposure** | **Outcome not present at start** | **On…(most important factor)** | **On other factors** | **Assessment of outcome** | **Long enough follow-up (assesed based on the specific study)** | **Adequacy (complete-ness) of follow-up (>90%)** | \| 9x \| \| --- \| |
| Alhamdan F. et al. |  |  |  |  |  |  |  |  |  | \| 6x \| \| --- \| |
| Go H. et al. |  |  |  |  |  |  |  |  |  | \| 7x \| \| --- \| |
| Goetzl L. et al. |  |  |  |  |  |  |  |  |  | \| 5x \| \| --- \| |
| Lal C.V. et al. |  |  |  |  |  |  |  |  |  | \| 9x \| \| --- \| |
| Miranda J. et al. |  |  |  |  |  |  |  |  |  | \| 9x \| \| --- \| |
| Murphy C.A. et al. |  |  |  |  |  |  |  |  |  | \| 7x \| \| --- \| |
| Ohta M. et al. |  |  |  |  |  |  |  |  |  | \| 8x \| \| --- \| |
| Ransom M.A. et al. |  |  |  |  |  |  |  |  |  | \| 5x \| \| --- \| |
| Spaul R. et al. |  |  |  |  |  |  |  |  |  | \| 6x \| \| --- \| |
